# Agroecology research in Europe: current status and perspectives

**DOI:** 10.12688/openreseurope.15264.1

**Published:** 2022-12-15

**Authors:** Ileana Iocola, Corrado Ciaccia, Luca Colombo, Baptiste Grard, Stefania Maurino, Alexander Wezel, Stefano Canali

**Affiliations:** 1 Council for Agricultural Research and Economics, Research Centre for Agriculture and Environment, CREA-AA, Rome, 00184, Italy; 2 Italian Foundation for Research in Organic and Biodynamic Agriculture, FIRAB, Rome, 00159, Italy; 3 Agroecology and Environment Research Unit, ISARA, Lyon, 69364, France

**Keywords:** transformation of agri-food systems, agroecological research, funding programme, research policy

## Abstract

**Background:** Agroecology is increasingly seen as an important contribution for the development of more sustainable agriculture and food systems. Research can have a main role to support this path. Although it seems that there is a gradual increasing body of agroecology research, it clearly lacks good knowledge about this. The main objective of this study was therefore to map research projects related to agroecology in Europe with the aim to characterize European research in terms of implementation of agroecology elements, identify needs for the future cross collaboration between countries and networks, and support the setting up of appropriate research agendas fostering agroecology research in Europe.

**Methods:** A desktop research with different databases related to European and transnational funding programmes was carried out to identify funded research projects involved in agroecology. The obtained projects were used to perform a social network analysis. Furthermore, two surveys were conducted, one with coordinators of identified projects and a second one for researchers engaged in agroecology.

**Results:** Our study highlighted a predominant trajectory of agroecology research prone to the transformation of the agri-food system. France, Germany, Italy, Netherlands, Portugal, Spain, and United Kingdom were the most active countries engaged in agroecology research. In all surveys, issues related to efficiency improvement, synergies strengthening, local economy development, and co-creation and sharing of knowledge were the most implemented to support agri-food transformation. Transdisciplinary approaches were mainly addressed by European projects. Surveys reported a limited participation of value chain actors, while researchers, farmers, and their associations were the most involved. Survey respondents suggested to increase project duration and to introduce flexibility methods to modulate research actions according to the dynamic of the contexts.

**Conclusion**: On the basis of the results, some policy recommendations were provided to fostering agroecology research in Europe and its contribution for transformation of agri-foods systems.

## Plain language summary

Agroecology aims to transform the agri-food systems by maximising ecological processes and increasing their environmental and socio-economic sustainability. In our study we analysed the role of current research related to agroecology carried out in Europe. We have found that aspects such as the improvement of efficiency, the enhancement of positive interactions among the different components of the agroecosystem, the development of local economy, and the sharing of the knowledge among farmers, researchers and other involved actors were the most implemented strategies in the research to support the agri-food system transformation. On the basis of our results, we have provided some recommendations to foster the contribution of research in the sustainable transformation of agri-food systems to policymakers who are responsible for the design and funding of research programmes related to agroecology and sustainable agriculture.

## Introduction

Agroecology is a multi-faceted approach to redesigning agri-food systems, with the aim to achieve environmental, socio-economic, and governance sustainability. Through transdisciplinary, participatory and change-oriented research and actions, agroecology is based on an integration of scientific disciplines, agricultural practices, and social movements ( Wezel *et al.*, 2009), and with a focus on social change ( Anderson *et al.*, 2021; Gliessman, 2016; Mendez *et al.*, 2013). Its definitions, scales, and dimensions have changed and been enriched over time ( Wezel & Soldat, 2009; Wezel *et al.*, 2020). At present, agroecology goes beyond the farm and the agroecosystem ( Gliessman, 2014); embracing the whole food system described by Wezel *et al.* (2016) as “a socio-technical network linking people, natural elements, and artifacts that interact with food issues”. Agroecology aims to achieve transformations in food systems, promoting a holistic and sustainable approach to food production reliant on place-based food interactions, food sovereignty, local knowledge and identity, and social justice ( Altieri & Toledo, 2011; Rosset *et al.*, 2011). From its origins, as a branch of agricultural or ecological sciences, agroecology currently addresses questions related to political and social disciplines. It represents a collective action mode for transforming the dominant agri-food regime and creating alternatives ( Levidow *et al.*, 2014) towards a process of redesigning food systems to achieve ecological, and socio-economic sustainability ( Gliessman, 2016).

In contrast to this transformative agenda, agroecology has been adopted by actors who promote conventional agriculture and the agro-industrial productivist model ( Holt-Gimenez & Altieri, 2013), through conservation agriculture and sustainable intensification approaches geared towards the increase of productivity. Such an orientation of agroecology remains embedded in the dominant agri-food regime, legitimising a biotechnological paradigm, and its potential to address environmental harms associated with industrial agriculture is questionable ( Alonso-Fradeyas *et al.*, 2020).

According to these two visions (transformative *vs*. conformative), the agroecology can result in different outcomes and socio-technical dynamics, influencing how science is conceived and articulated. Amongst the broad range of topics identified in European agroecological research ( Wezel *et al.*, 2018), some research approaches are organised in order to integrate the participation of several academic (with different skills and expertise) and non-academic actors (farmers, consumers, value chain actors, policy makers, *etc*.) and to promote a wider territorial development, while others are developed more in line with the dominant agri-food regime and adopt unidirectional forms of knowledge transfer and technology.

This interest in agroecology did not emerge simultaneously and with similar intensity in the different European territories and countries. Under the initiative of Food and Agriculture Organisation (FAO), France, Italy, Hungary, and Switzerland joined the so-called group of the Friends of Agroecology, manifesting an explicit intention to support international efforts in this direction ( Bruil *et al.*, 2019). These countries, acting as agroecological forerunners at European level, might have gained a leading position in promoting agroecology, imprinting different visions and towing the European research and innovation communities.

Analytical distinctions are necessary to identify research elements in agroecology characterized by their transformative potential. This will enable the setup of appropriate research agendas supporting the transformative direction. With this in mind, the main objective of this study was to map research projects related to agroecology in Europe with the aim to: i) characterize EU research in terms of implementation of agroecology elements and evaluate if the current agroecology research in Europe really contributes to create transformative alternatives to the current agri-food regime; ii) understand connections among European countries and identify the most powerful and influential countries investing in agroecology research; and iii) provide recommendations for future research agendas to better strengthen agroecology and its transformative role in Europe.

## Methods

### Research project mapping

Desktop research was carried out from March to May 2021 to identify research projects involved in agroecology in Europe starting from 2014 onwards (according to the Horizon framework). We did this by consulting the main relevant available databases on funded projects belonging to two different categories: a) European (EU) research projects financed by funding programmes for research framed and funded by European Union (i.e., work programmes of the Horizon framework); b) Transnational research projects financed by funding programmes for research co-framed and co-funded by Member States with the participation of European Union (i.e., public to public partnerships -P2Ps in the past Horizon framework and European Partnerships of Horizon Europe). For the EU category, the CORDIS database and EURAKNOS thematic networks were consulted.

With the aim to include EU projects where the term “agroecology” was explicitly mentioned, we used the following truncated keywords to perform our preliminary research in the CORDIS database: agroecolog* (to include both agroecology and agroecological) and agro-ecolog* (for agro-ecology and agro-ecological). However, in order to include more projects that did not explicitly mention agroecology but that could be referred to agroecologically linked approaches and systems, we considered the additional following keywords: *agroforestry, silvopasture, silvoarable, food justice, food system, territorial food system, food sovereignty and rural development*. Regarding the EURAKNOS thematic networks, since it is made up of only 35 projects, no keywords have been searched but all the projects were investigated.

For the transnational research projects, we consulted the ERA-LEARN and Organic e-prints databases. Since it was not possible to search by keywords in ERA-LEARN database, all the 34,400 projects funded within the Horizon 2020 framework were checked. Only three projects were instead obtained from Organic e-prints after setting the filters relating to the years (2014 or later) and the English language. All identified projects were analysed using information available on their websites. Only projects where agroecology was explicitly mentioned in the project narrative, or the projects that were characterized by actor engagement and addressed the criteria of at least level three (redesign the agroecosystem based on ecological processes: e.g. enhancing ecological function and ecosystem services; increasing agrobiodiversity; improving resilience of farming system to some specific disturbances) of the framework proposed by Gliessman (2014) for classifying food system change were taken into account. For the project selection, we considered the list of criteria of transition reported in the Excel file of the Agroecology Criteria Tool (ACT) methodology which were based on the work carried out by DeLonge *et al.* (2016) and were already embedded within the five levels of Gliessman’s framework. Projects covering at least one criterion of the level three, or level four or level five of the framework were considered in our study. No automation tools were used in the process. Four people from two different institutions worked together on this issue to avoid bias due to any personal judgments and interpretations. A provisional list of selected projects was also shared in October 2021 and discussed for its validation with partners of AE4EU project. A further validation was performed by checking if projects identified by the Standing Committee on Agricultural Research - Strategic Working Group on Agroecology (SCAR-AE) as relevant for agroecology and presented in four slam sessions (from March to October 2021) were also included in our list. All the 16 projects presented by SCA-AE were in our list thus confirming the validity of our methodology.

The list of the identified projects was included in a database ( Iocola *et al.*, 2022a). We decided to include in it information that was easily available on websites and useful for identifying projects (project acronym and title, start and end dates, main objectives, source of funding, total costs, and available URL), contacting project coordinators (coordinator name and email address), and identifying research collaborations among countries (participating partners and their related countries).

### Social Network Analysis

With the aim of understanding and measuring relationships among European countries involved in agroecology research projects, a social network analysis (SNA) was conducted, considering all the identified research projects using the package igraph ( Csardi & Nepusz, 2006) of R software, version 4.1.2. In order to focus the analysis on the European region, the SNA included the countries considered part of Europe by taking into account only the members of the Council of Europe. In the SNA, each node represents a country and there is an edge between two nodes when the two countries were partners in the same project. Edges were weighted according to the times two countries cooperated together for a project.

The following metrics were used in the SNA study: a) the density (the ratio of the number of edges and the number of possible edges). The value of this index vary from 0 to 1 which is obtained when there is at least one connection between all the nodes of the network; b) the diameter (the length of the longest path - in number of edges -between two nodes); c) the mean distance (the average number of edges between any two nodes in the network); d) degree centrality (DC) which reflects the direct relational activity of a node by measuring the number of direct connections each node occupies in a relationship ( Wasserman & Koehley, 1994), and e) closeness centrality (CC) which measures how close a node is to the other nodes in the network ( Borgatti & Everett, 1997). It is defined by the inverse of the average length of the shortest paths to/from all the other nodes in the graph. Weights were also used for calculating weighted shortest paths. The greater the weight, the shorter the distance among nodes.

Lastly, we used the clustering optimal function of igraph to detect the presence of communities (also called groups, clusters, or modules) among countries involved in agroecology research projects. This function calculates the optimal community structure for a graph by maximizing the modularity score over all possible clusters of all sizes ( Brandes *et al.*, 2007). Modularity measures the strength of division of a network into communities. It is defined as the fraction of the edges that fall within the given groups minus the expected fraction if edges were distributed at random in an equivalent network. The modularity can be either negative or positive (up to 1). It is positive if the number of edges within groups exceeds the number expected on the basis of chance thus indicating the presence of a community structure.

### Surveys

Two different online surveys, containing both multiple-choice and open-ended questions, were conducted to better understand how agroecology is perceived and implemented by research planners in Europe. One survey was for the coordinators of the research projects identified in the mapping activities (see Underlying data - 3). The coordinators were contacted by email, and they were asked to fill in a questionnaire on Google Form. The questionnaire ( Iocola *et al.*, 2022c) was organized into three sets of questions aimed at acquiring information on: 1) a general overview of the project and the used research approaches; 2) the actors involved in the projects, in which stages they are engaged, and methods and learning processes implemented to facilitate participation; and 3) main lessons learned and challenges addressed by the project. Furthermore, the Excel file of the Agroecology Criteria Tool (ACT) was sent to the potential respondents as attachment to be filled in together with the questionnaire but as optional.

The second survey was designed for researchers from public and private research institutions in Europe involved in agroecology. No participant selection or exclusion criteria were used for this survey. The goal of the survey was to collect information from respondents on their experiences in agroecology research and to compare their answers with those obtained from the previous survey where respondents were selected by our methodology. A second questionnaire ( Iocola *et al.*, 2022d) was prepared for this survey. The link to the Google form was widely disseminated through public and private contact mailing lists, networks, and websites (such as AE4EU project, ISOFAR). The questionnaire was articulated in three sets of questions related to: a) experiences on agroecology research funding; b) experiences on agroecology research and actor engagement; c) individual background information. In this survey, a five-point Likert scale was used to ask the perceived importance of some themes and approaches for agroecology, where five represented the most important for the respondents and 1 the least relevant. From the values of the five-point Likert scale (1–5), the mean and the standard error of responses were also calculated.

The two different questionnaires were structured in order to be able to capture some relevant and conflicting issues and approaches in agroecological research, as highlighted by many authors, such as: preference of sustainable intensification approach *vs*. eco-functional intensification ( Levidow *et al.*, 2014), conventional *vs*. organic agriculture ( Buttel, 2003; Levidow *et al.*, 2014), use of genetically modified organisms (GMOs) ( Bonny, 2017; Giller *et al.*, 2017; Lotz *et al.*, 2020), multidisciplinary *vs*. interdisciplinary ( Mauser *et al.*, 2013; Popa *et al.*, 2015); actors diversity and their degree of engagement in the process of knowledge/transdisciplinarity ( Schneider & Buser, 2018); and the presence of living labs and use of research infrastructure ( McPhee *et al.*, 2021). In order to avoid bias in the answers especially in controversial topics, an impartial and non-judgmental style was used in closed question. Moreover, these questions were generated to be equal in desirability.

In both surveys, we investigated the interaction with non-academic actors in the co-creation of knowledge using a classification proposed by Schneider & Buser (2018). These authors identified six different degrees of interaction describing different modes of non-academic actors’ involvement in research and what roles are attributed to them ( Table 1). The degree of interaction was identified in three different phases of the research ( Lang *et al.*, 2012): 1) ‘‘Framing the problem and research goal’’, where the most relevant problems addressed by research are defined; 2) “Knowledge production”, where the new knowledge is produced; and 3) ‘‘Bringing results to fruition’’, where the new knowledge is re-integrated into scientific and societal practice.

**Table 1.** Different degrees of actor interaction over the three phases of research. Interaction degree ranges from 1=low to 6=high. Source: Schneider & Buser (2018). Reproduction of tables from any SpringerOpen article is permitted without formal written permission from the publisher or the copyright holder.

Interaction degree		Problem-framing and goal- definition phase	Knowledge-production phase	Bringing-new knowledge to fruition phase
Co-production	6	Problem and goal co-framed by scientists and stakeholders; main elements of the proposal arevcodesigned	Co-production of knowledge including deliberation and integration of all relevant stakeholder perspectives regarding main project elements	Co-producing main project outcomes and jointly constructing follow-up structures/actions, and engaging in societal learning processes
	5	Problem and (overall) goal co-framed by scientists and stakeholders; some elements of the proposal are codesigned	Co-production of knowledge including deliberation and integration of all relevant stakeholder perspectives regarding some project elements	Co-producing some project outcomes and/ or jointly constructing follow-up structures/ actions, and/or engaging in societal-learning processes
Consultation	4	Problem and goal framed by scientists; broad consultation of stakeholders leading to minor thematic adjustments of the proposal dealing with different stakeholders’ perspectives and priorities	Knowledge production by scientists, taking into account various stakeholders’ knowledge and perspectives. A wide range of stakeholders are consulted, but the knowledge is structured according to the scientists’ concepts	A wide range of stakeholders is consulted to discuss research results. The stakeholders’ perspectives influence final interpretations and recommendations
	3	Problem and goal framed by scientists; consultation of some stakeholders leading to minor thematic adjustments of the proposal	Knowledge production by scientists; some key stakeholders are informed and consulted for fine-tuning	Stakeholders are informed and final results and recommendations are jointly discussed
Informing	2	Problem and goal framed by scientists; a few stakeholders are informed about the project and feedback is encouraged. Stakeholder interactions influence logistical issues, but not project goals	Knowledge production by scientists; some stakeholders are informed and given an opportunity to provide feedback, e.g. in individual meetings, but they have hardly any influence on knowledge production	Stakeholders are informed about final results by means of articles and at meetings that offer a chance to clarify questions
	1	Problem and goal framed by scientists; a few stakeholders are informed about the project. Stakeholder interactions do not influence the proposal	Knowledge production by scientists; some stakeholders are informed about the status of the project	Stakeholders are informed about final results by means of articles in professional journals or newspapers

Respondents of both surveys were also asked to identify how their research or project support agri-food system transformation through agroecology in Europe according to a set of issues ( Table 2) proposed by the ACT methodology and related to the FAO’s “10 elements of agroecology” ( FAO, 2018). These elements are embedded by ACT within the five levels of food system transformation levels proposed by Gliessman (2014).

**Table 2.** List of issues to support agroecology transition of agri-food systems. Issues are taken from the Agroecology Criteria Tool (ACT) methodology ( https://www.agroecology-pool.org/methodology/) and embedded within the FAO elements of agroecology ( FAO, 2018) and the 5 levels of food system transformation proposed by Gliessman (2014).

Gliessman’s levels	FAO elements	Issues
Level 1	Efficiency	Improving approaches focused on increasing/maintaining yield and reducing external input use
Level 2	Recycling	Strengthening practices that close cycles, drive the recycling of nutrients, biomass, and water within production systems
	Regulation and balance	Optimizing the biophysical mechanisms and interactions within farming systems to boost natural regulation processes, including pest regulation, and to temper disturbances through alternative practices that substitute toxic inputs
Level 3	Synergies	Carefully designing diversified system and integration of elements in the system to optimize biological synergies
	Diversity	Optimize the vertical, temporal, spatial diversity of species and genetic resources
	Resilience	Increasing the capacity to recover from disturbances including extreme weather events
Level 4	Circular and solidarity economy	Reconnecting producers and consumers, prioritizing local markets and short food circuit, and supporting local economic development
	Healthy and cultural food	Supporting healthy food production and consumption, and cultural identity tied to landscapes and food systems
	Co-creation and sharing of knowledge	Promoting innovation co-created through participatory processes and context-specific knowledge
Level 5	Human and social value	Improving rural livelihoods, equity, and social well-being (dignity, inclusion, and justice) by building autonomy and adaptive capacities
	Responsible governance	Promoting responsible, effective, transparent, accountable, and inclusive governance mechanisms at different scales

These data, together with the Excel file filled in by the project coordinators, other answers and documents available on the websites of the projects were used to identify the research’s actions according to the five levels of Gliessman. On this basis, projects and studies carried out by the respondents of the second survey were classified in the four categories proposed by the ACT tool methodology ( Pavageau *et al.*, 2020): a. Incremental change with projects/research addressing solely the level one1 and/or two; b. Agroecological transformation where projects/research are also engaged with level three; c. Systemic where in addition to level three, level four and/or five are also addressed; and d. Social enablers with the engagement only with level four and/or five.

## Results

### Social network analysis

**Figure 1.**
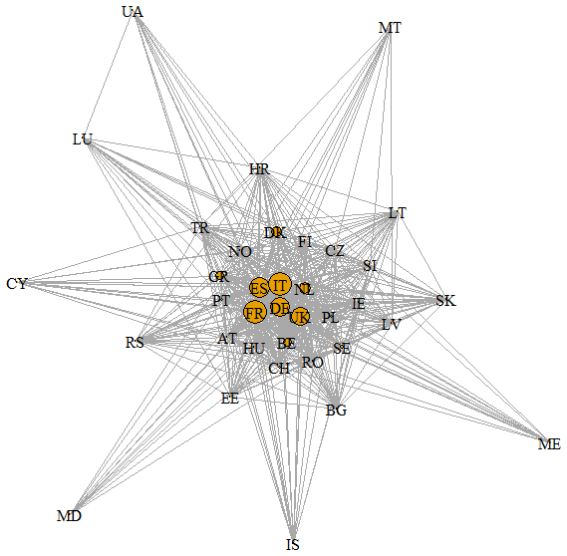
Social Network analysis at country level on agroecological research projects. The codes of the different countries are reported in the nodes of the network (AT-Austria; BE- Belgium; BG-Bulgaria; CH- Swiss; CY- Cyprus; CZ- Czech Republic; DE-Germany; DK-Denmark; EE-Estonia; ES-Spain; FI- Finland; FR-France; GR-Greece; HR-Croatia; HU- Hungary; IE-Ireland; IS-Iceland; IT-Italy; LT- Lithuania; LU- Luxembourg; LV-Latvia; MD- Moldova; ME-Montenegro; MT-Malta; NL- Netherlands; NO- Norway; PL-Poland; PT- Portugal; RO-Romania; RS-Serbia; SE- Sweden; SI-Slovenia; SK- Slovakia; TR- Turkey; UA- Ukraine; UK- United Kingdom)

A total of 124 agroecology projects were identified in the mapping analyses divided between 68 EU projects and 56 transnational projects ( Iocola *et al.*, 2022a). The social network ( Figure 1, Iocola *et al.*, 2022b) obtained with the identified projects resulted in 36 nodes (or countries) and 486 edges. In the figure, the node size is proportional to the number of research projects coordinated by each country, while the edge line thickness is proportional to the weight (w) of the edge. The SNA density was 0.77, its diameter was only two, while the mean distance was 1.23. France (n=18), Italy (18), Spain (16), Germany (15), United Kingdom (14) were the countries that coordinated a greater number of projects, with a strong predominance of the transnational projects for Italy (15) and EU projects for France and United Kingdom (respectively, 13 and 14). In Spain and Germany, the quantity is instead well divided between European (eight for Spain, seven for Germany) and transnational projects (eight for both the countries). 

**Figure 2.**
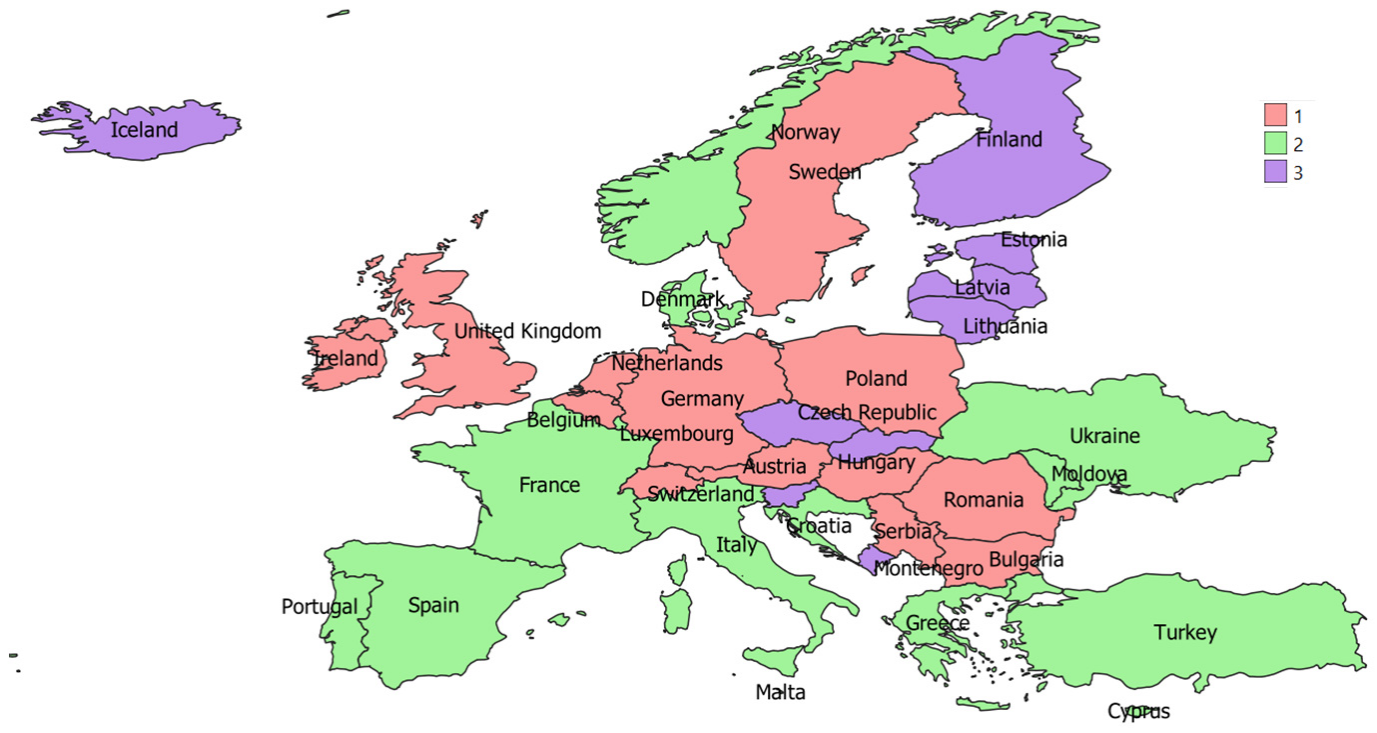
The three groups of countries obtained in the social network analysis.

Italy and France exhibited more important numbers of cooperation (w=70), followed by Italy and Spain (67). On the contrary, there were 106 interactions between countries out of 486 characterized by just one cooperation. Spain (DC=35), Italy (35), France (34), Netherlands (34), and Portugal (34) had the highest degree centralities, having established direct relations with many countries of the network. On the contrary, Ukraine (12), Moldova (11) and Malta (11) were the countries characterized by fewer direct interactions. All nodes showed a similar score for the Closeness Centrality going from the highest value (CC= 0.016) for Iceland, Luxembourg and Ukraine to the lowest one (0.07) for Belgium. France (0.0138), Netherlands, Germany, Spain, Italy, and Portugal (all with 0.0137) also continued to perform well in this index. Lastly, three different communities of countries with a maximum modularity of 0.02 were identified by our analysis. The three groups are reported in Figure 2.

### Survey analysis

We received 26 replies ( Iocola *et al.*, 2022c) out of 124 submissions (a total of 20.6 % of responses) from the coordinators of the identified projects: 17 for the EU and nine for the transnational ones. Moreover, some of the respondents (seven EU and five transnational) filled in and sent us the optional Excel file of the ACT.

Considering the survey open to all researchers involved in agroecology, we received 35 responses ( Iocola *et al.*, 2022d) from eight Countries (see Underlying data - 4): one from Estonia, four from France, one from Georgia, two from Germany, three from Greece, 19 from Italy, two from Spain, and three from the United Kingdom. Most respondents (63%) were over 50 years, 29% were aged between 35 and 50, and only 9% were under 35. Respondents varied widely in the number of years they have been working in agroecology: 54% of respondents were in later career stage in agroecology (> 10 years), 23% in the middle (5–10 years), and 23% in earlier stage (<5 years). Most of the respondents (83%) were related to agronomic sciences (in particular soil sciences and plant pathology), 11% were from economic sciences, and 6% from social science. 89% work in public research organisation or academia/university, 11% in private research or non-governmental organisations.

***General issues and approaches in agroecology research***. EU projects presented a longer average duration (3.7 ± 0.9 years) than transnational projects (2.8 ± 0.7 years). Regarding the geographic (local, regional, national, international) scales addressed simultaneously by a project, EU projects showed an average of three scales with the highest values achieved by the international (addressed by 94% of the projects) and local (76%) scales, followed by the regional (71%) and the national ones (65%). Transnational projects addressed simultaneously less scales (average of 1.5) with the highest values showed by the international (78%) and the regional scales (44 %) followed by national and local (both 33%). The different sustainability pillars (environmental integrity, economic resilience, social well-being, good governance) defined by Sustainability Assessment of Food and Agriculture systems (SAFA) were taken into account by the various research projects with the highest percentages achieved by the environmental (94% for EU, 89% for transnational) and economic dimensions (82% for EU, 78% for transnational). The social pillar was covered only by 59% of EU projects and 44% by transnational, and governance by 59% EU and 33% transnational. 

Genetic modification techniques (Genome editing, Cisgenic and Transgenic modifications) were implemented in none of the projects, whereas in only five (three EU, two transnational) traditional modification such selective breeding and crossbreeding approaches were applied. Regarding organic and conventional production systems, our findings indicate both were simultaneously taken into account by the majority of EU research projects (82%), but less with 44% for transnational. There were also some research projects that only dealt with the conventional system (6% for EU, 11% for transnational) or with organic agriculture (12% for EU, 44% for transnational). 53% of EU and 56% of transnational projects were dealing with the use of synthetic inputs. The sustainable intensification approach (defined in the questionnaire as “Agricultural yields are increased without adverse environmental impact and without the conversion of additional non-agricultural land *”* according to FAO, 2011) was selected by only one EU project, whereas ecological intensification (“Agricultural system performances are improved through a knowledge-intensive process that requires optimal management of nature’s ecological functions and biodiversity *”;* FAO, 2011) was chosen by all others.

Likewise, the definition of the integration of scientific disciplines using an interdisciplinary approach (“Project is composed by members with different expertise covering different fields and disciplines. They collaborate and share ideas from the beginning to resolve issues”) was selected and implemented by most of the projects. Instead, the multidisciplinary approach (“The project is composed by members with different expertise covering different fields and disciplines. Each member works autonomously to come up with findings. Result sharing allows to see whether the different findings are consistent or contradictory *”)* was implemented in only one EU project.

Living labs (LLs) (defined in the questionnaire as “user-centred, open innovation ecosystems based on systematic user co-creation approach, integrating research and innovation processes in real life communities and settings. In simpler terms, living labs are initiatives in which experimentation is conducted on real farms, in specific territorial and community contexts, with farmers and other actors involved from the beginning as equal partners in proposing ideas, testing them, improving them and promoting them further *”*) were present in 53% of EU and 22% of transnational projects, while Research Infrastructures (RIs) (“Research infrastructure refers to the facilities, resources and services that are used by the research and innovation community to conduct research and foster innovation in their fields. Research infrastructure includes: major research equipment, knowledge-based resources such as collections, archives and data, e-infrastructure such as data and computing systems and communication networks”) were used/developed by 18% of EU and 44% of transnational projects. RIs remained available even after the end of the projects for 86% of cases and of these, only a third (33%) consisted of databases made available for other future research.

**Figure 3.**
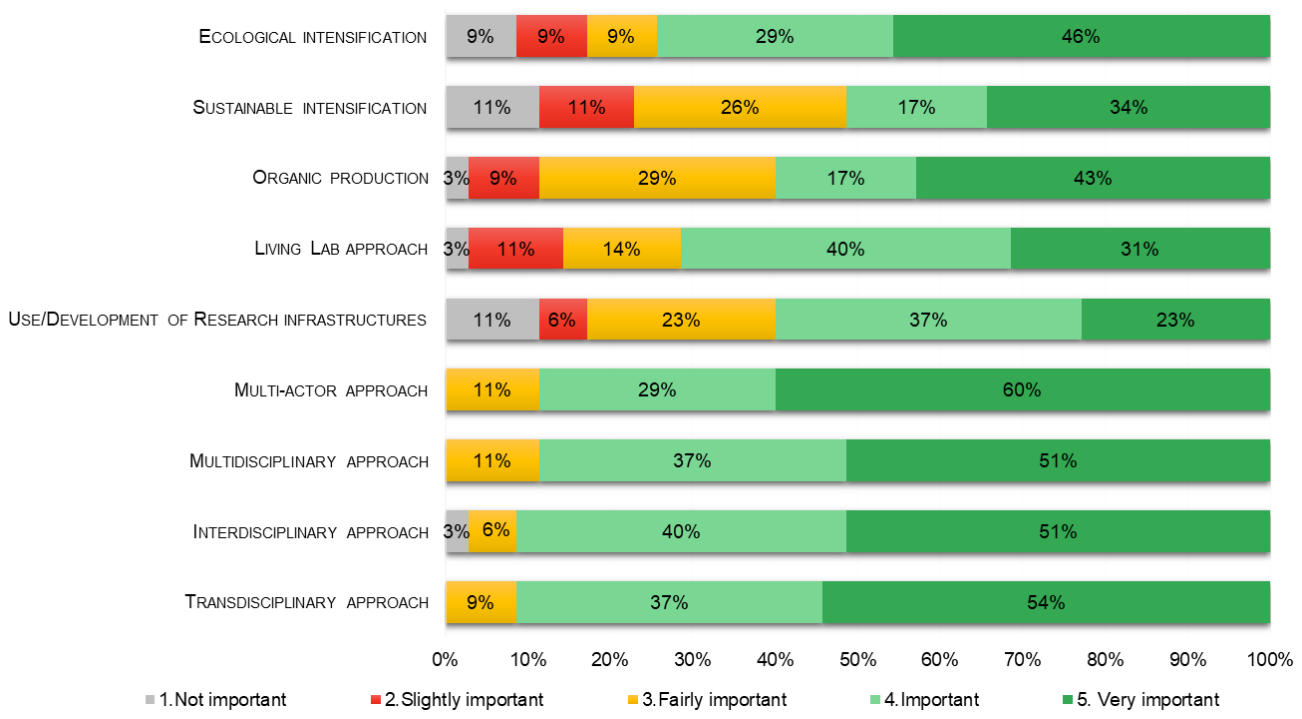
Percentages of importance of themes and approaches in agroecology as reported by respondent researchers (n=35).

The degree of importance of some of the above issues was also explored in the survey for agroecology researchers ( Figure 3). Ecological intensification showed a very slightly higher degree of preference (mean= 3.9 ± 0.22) than sustainable intensification (3.5 ± 0.23), while organic production was considered very important for agroecology by 43% of respondents reaching a mean value of importance of 3.9. LLs (mean= 3.9 ± 0.18) and RIs (3.5 ± 0.21) were also considered quite important to support research in agroecology. No substantial difference was evident between multidisciplinary (mean= 4.4 ± 0.12) and interdisciplinary (4.4 ± 0.14), while the importance for respondents of actors engagement in agroecology was shown by the highest means and lowest standard error values obtained by transdisciplinary and multi-actor approach both with a mean of 4.5 and a standard error of 0.11. Moreover, most of the agroecology studies carried out by respondents in this survey concerned field (71%), farm (83%), and territorial geographic scales (63%), while the value chain was more rarely addressed (14% for upstream, 14% for downstream, and 11% for the whole value chain).

**Figure 4.**
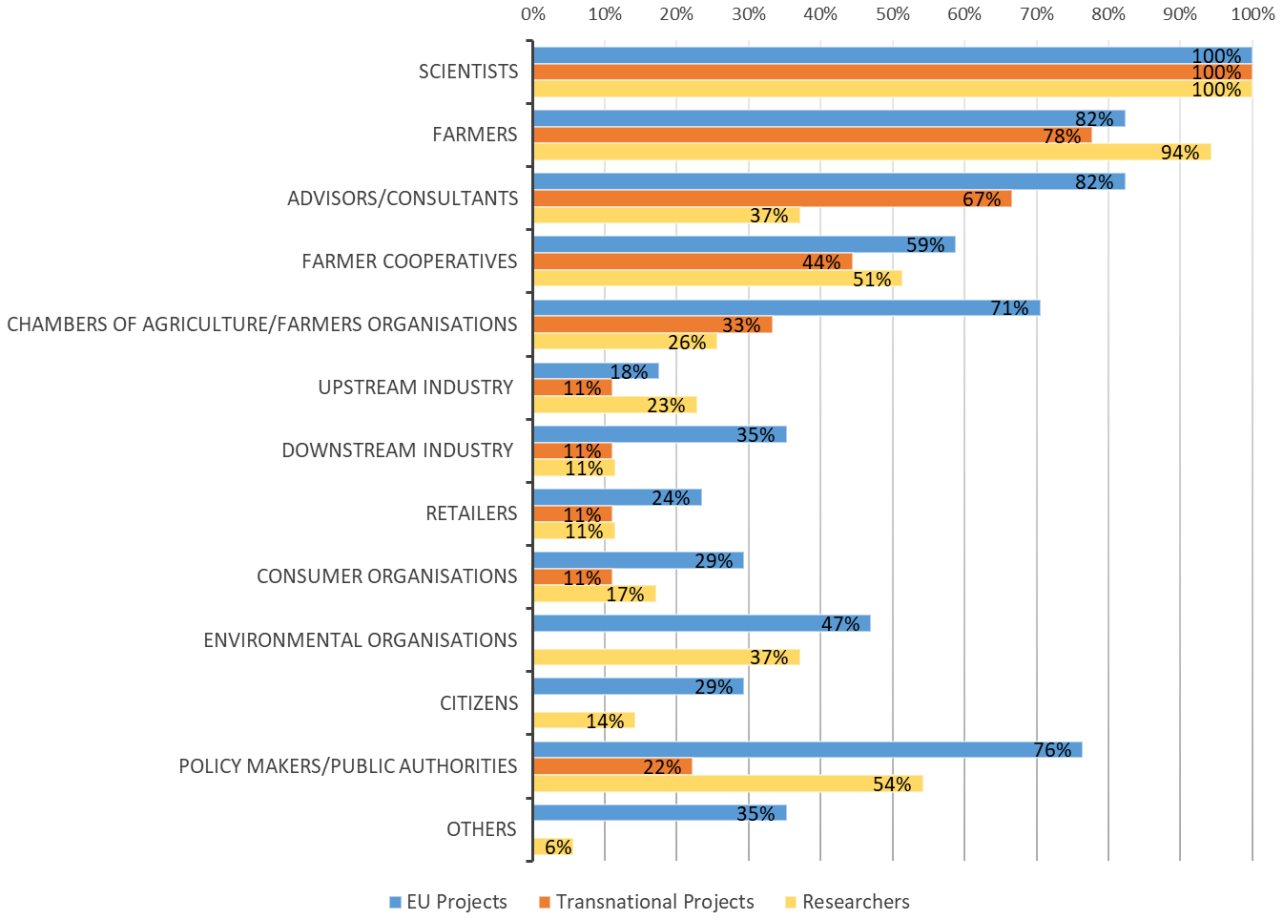
Diversity of actors and their percentages of engagement in agroecology research. EU: European projects, Transnational: Transnational projects, Researchers: research carried out by researchers involved in agroecology. Others for EU projects: *Community seed banks, seed savers, bioenergy producers, NGOs providing access to land, ecosystem service users in the local community, Education organisation, Statutory levy board for agricultural development*. Others for Researchers: *Professional organisations (organic assoc., breeding assoc. etc)*

**Figure 5.**
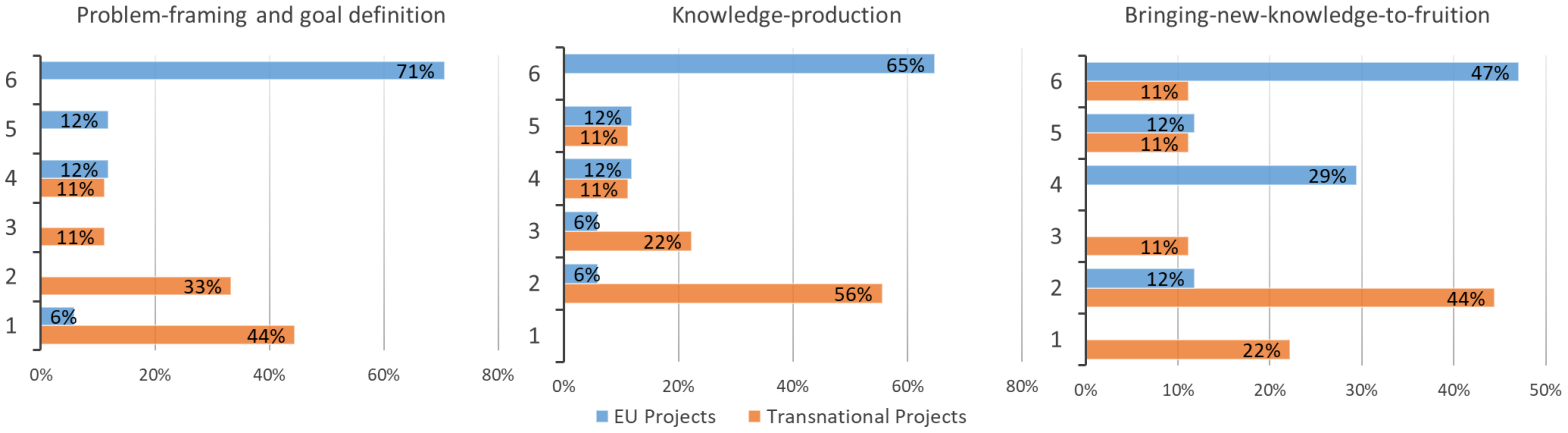
Degrees of actor interaction over the three phases of research in agroecology projects. Interaction degree ranges from 1=low to 6=high. EU: European projects, Transnational: Transnational projects.

***Actor engagements***. The average number of types of actors involved in agroecology projects was seven for EU projects, and four for transnational projects. Beside scientists, the actors mostly involved in the projects were farmers, their associations, and cooperatives as well as advisors ( Figure 4), while there was limited engagement of upstream and downstream value chain actors. Environmental organizations, citizens, and policy makers were mostly present in EU research projects. On average, the different types of involved actors in the studies carried out by researchers involved in agroecology was quite high (n=7) and the obtained percentages were totally in line with the responses provided in the previous survey by the projects’ coordinators. In fact, this survey also confirmed a limited participation of value chain stakeholders. Considering the degrees of actors’ interaction ( Table 1) over the three phases of a research project ( Figure 5), the higher degrees, characterized by modes of collaboration in which knowledge is truly co-produced and the research process is co-shaped with non-academic actors, were higher in EU projects in all the phases of the research. On the contrary, transnational projects showed the highest percentages in t lower degrees of interaction, limited to information-sharing or actors’ consultation. Furthermore, selecting only the projects with the presence of LLs (9 EU and 2 transnational), the higher degrees of actor interaction (≥ 5) during the three phases were almost exclusively found with EU projects (problem framework: 89% EU, 0% transnational; knowledge production: 89% EU, 0% transnational; new knowledge to fruition: 67% EU, 50% transnational). However, although truly co-learning processes were not implemented in all agroecology projects, coordinators were aware of the importance of strengthening agroecology research impact. In fact, many lessons learned reported in the survey underlined these aspects while considering difficulties that still exist in implementing these approaches (quotes 1–5) and in reaching a diversity of actors (quotes 6–7) ( Table 3).

**Table 3.** Lessons-learned and comments reported in the survey for coordinators of agroecology research projects.

n.	*Quotes*
1.	*“Great care may need taken to help ensure a ″better balance″ of science/academic goals with the transdisciplinary and business* * elements. Many academics, simply, do not understand what transdisciplinary is, and how this can help direct their research effort* *towards being more impactful” - EU*
2.	*“Involvement of stakeholders in co-creation is useful and facilitates the impact but it is at the same time very challenging due to the* * differences in understanding how the science is run”-EU*
3.	*“Researchers′ and scientists′ targets and how their work is evaluated is in contrast with the type of work and activities needed to engage* * effectively with stakeholders. The importance of academic publications is still too great to allow scientists to dedicate more time to * *stakeholder engagement” –* Transnational
4.	*“Projects are difficult when trying to be truly collaborative, but we learned a lot and dissemination and collaboration with stakeholders* * becomes stronger” -*Transnational
5.	*“Agroecology has a cultural dimension. Scientist and stakeholders have to change their mind/way of thinking regarding their * *relationships with living beings and environment”- EU*
6.	“ *Importance of considering actors who are hard to reach to ensure Multi-Actor Platforms reflect the principals of just transitions.”- EU*
7.	“ *Stakeholders and strategies for further enhancement and contributions to transitions to agroecology require flexibility to compositions* * and the addition of further actors to the network - EU*

EU: European project; Transnational: Transnational project.

In the survey open to agroecology researchers, farmers and other non-academic actors involved in the research were mainly identified by previous collaborations (83%), by a request coming from the same actor (54%) or through a stakeholder analysis (43%). Considering the three phases of the research, interaction with farmers and other non-academic actors mainly took place in phase one (Problem definition: 71% for farmers, 57% for other non-academic actors) than in the other two phases (Knowledge production: 46% for farmers, 51% for other non-academic actors; New knowledge to production: 63% for farmers, 49 % for other non-academic actors). Moreover, while farmers were involved in all the studies carried out by the respondents, other non-academic actors were implicated in 94% of the cases. Monitoring and evaluation of engagement of farmers and other non-academic actors throughout the duration of the research was carried out only by respectively 46% (for farmers) and 31% (for others) of the respondents. Monitoring and evaluation were mainly carried out through periodical meetings, participative workshops, evaluation surveys, periodic contacts and self-reflection. In general, the willingness of farmers (mean= 3.5 ± 0.18) and other non-academic actors (3.4 ± 0.17) to participate to the research was considered somewhat satisfactory by researchers, while the level of satisfaction regarding rewards/promotion/recognition for this type of participatory research was slightly lower (2.5 ± 0.17). Many comments provided by the respondents highlighted the importance of engaging farmers and other food system actors in research related to agroecology, even if there are some difficulties and barriers, as participatory activities need time and energy and because of the low interest of actors not directly linked to the production (quotes 1–4 in Table 4).

**Table 4.** Lessons-learned and comments reported in the survey for researchers involved in agroecology.

n.	Quotes
1.	“ *Interest from stakeholders (particularly the not-farmer ones) is a crucial aspect, sensibilization activities should be implemented to * *increase stakeholders’ awareness they can become actors of the food systems*”
2.	“ *Collaboration with farmers need time and energy to be kept alive, this dimension of maintaining the collaboration "alive" and* * "dynamic" is often underestimated in the project funding and not rewarded in term of research*”
3.	“ *Involvement of farmers (and farmers interest to PAR activities) is easier the further away they are from large markets and the more* * remote the rural areas*”
4.	“ *Very difficult to engage actors not directly connected with the production*”
5.	*“More agroecology, longer projects, adding new partner every year, including small farmers as micro-business in Innovation actions,* *more social and food system aspects”*
6.	*“Projects with longer duration (over five years) in fact agro-ecological transition requires long term processes”*
7.	“ *Programmes are still too siloed: integration of all dimensions of agroecology are needed (environmental, social, economic, political - or* *across the various principles of agroecology, e.g. 13 HLPE principles)*”
8.	“ *Yield increase paradigm still too much in some calls”*
9.	*“Agroecology and ecological intensification need to be embedded into the one-health concept and clearly focussed”*
10.	*“Specific funding calls for agroecology are rare”*
11.	*“In agroecology one cannot ignore the needs of food system operators and consumers/civil society for which the bottom-up approach* *should be implicit. These needs should then be collected by researchers, representatives, and institutions who, together with operators* *in the food supply chains, consumers and other stakeholders, develop the research project, enriching it with any elements that may be* *overlooked and, in any case, shared by all”*
12.	*“Funding advocacy and implementation of input independence strategies throughout in the food systems, and particularly, the * *productive smallholder farming sector, should be a priority”*
13.	*“At a minimum, we need consultancy and technical assistance programs for businesses in the food supply chains, especially* *farmers and processors, to be implemented through the establishment of communities of practice and living labs, information and* *communication campaigns aimed at consumers to gain awareness of sustainable diets and consumption styles, their ability to induce* *changes in the production system in view of greater sustainability by interacting with producers”*
14.	“ *Deliverables, milestones and all of the like, are designed so a non-expert project evaluator (officer) can check the boxes and judge the* *good progress of the project. Its real impact, however, is another story”*
15.	*“To be funded, projects need more skills in writing and speaking bureaucratically language, than scientific skills and practical potential.* *Too many formalisms. Projects are written with the evaluation sheet in mind, not with innovation potential in mind. Projects are judged* *as good when they contain all the wizard words, rather than having real science and innovation potential.”*
16.	*“Less bureaucracy, more access to private companies”*
17.	*“More flexibility on how to spend the budget, based on need”*
18.	*“Programmes/calls should be build considering the possibility to enable the direct participation of stakeholders (e.g. farms,* *organization) as partners”;*
19.	*“Farmers engagement in the project need to have funds since they are key part of the project”*
20.	*“Individual projects should not become too big (more than 2–5Million) otherwise only the big organisations will be able to coordinate* *such a consortium. In contrast, smaller budgets would allow smaller organisations and consortia to profit from the programmes”*

**Figure 6.**
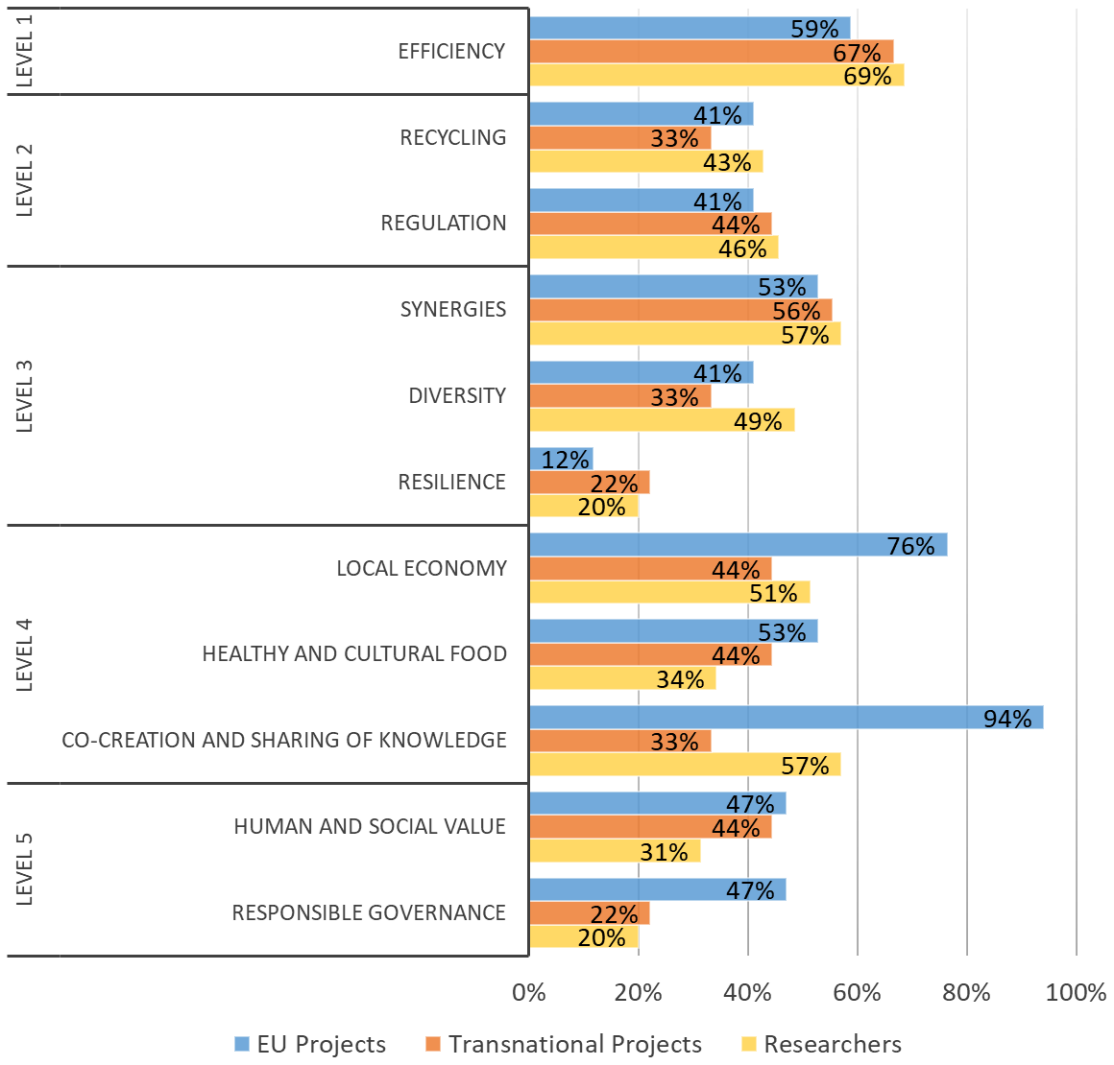
Percentages of the issues promoted by agroecology research to support agri-food transformation in Europe. Issues are related to the FAO elements of agroecology ( FAO, 2018) and embedded in the five levels of food systems transformation proposed by Gliessman (2014). EU: European projects, Transnational: Transnational projects, Researchers: research carried out by researchers involved in agroecology.

**Figure 7.**
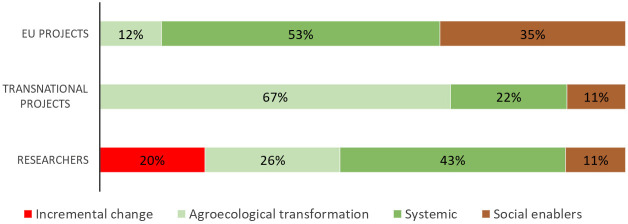
Classification of agroecology research. Projects and research carried out by researchers involved in agroecology were classified according to the four categories proposed by Pavageau *et al.* (2020): *Incremental change* with projects/research addressing solely the level 1 and/or 2; *Agroecological transformation* where projects/research are also engaged with level 3; *Systemic* where in addition to level 3, level 4 and/or 5 are also addressed; and *Social enablers* with the engagement only with level 4 and/or 5. EU: European projects, Transnational: Transnational projects, Researchers: research carried out by researchers involved in agroecology.

***ACT methodology***. Respondents of both surveys were also asked to identify how their research or project supported agri-food transformation through agroecology in Europe according to a set of issues ( Table 2, Figure 6). Considering all EU and transnational projects, the highest percentages of responses were obtained for issues related to the agroecology elements “efficiency” (62% of total projects), “synergies” (54%), “local economy” (65%), and “co-creation and sharing of knowledge” (73%). Results obtained from the researchers involved in agroecology ( Figure 6) were totally in line with those obtained by the projects’ coordinators. The highest percentages of responses were also obtained for Efficiency (69%), Synergies (57%), Local economy (51%), and Co-creation and sharing of knowledge (57%). The classification of projects and studies carried out by agroecology researchers according to the ACT methodology is reported in Figure 7. The highest percentage for EU projects is achieved by the “Systemic” category (53% of projects) while transnational projects were predominantly made up of the “Agroecological transformation” category (67%). The “Incremental change” addressing exclusively the level 1 and/or 2 of food system transformation was only present in the survey open to all agroecology researchers (20% of the cases).

***Focus on funding***. Some questions of the survey for researchers also addressed experiences on research funding in agroecology. Respondents obtained funding for their agroecology research principally from national (69%) and European (63%) funding programmes. Less funds derived from the transnational initiatives (31%). Researchers were asked the importance of some changes in programmes to better support research in agroecology according to a five-point Likert scale. “Recognition of a lump sum for costs incurred in stakeholder engagement and consultation during the co-framing of the proposal” (4.2 ± 0.14), “Introduction /presence of methods capable of guaranteeing the flexibility of project actions based on the dynamics of the contexts” (4.2 ± 0.15), and “Increase of the duration of the projects funded by the programme” (mean=4.2 ± 0.16) were the most relevant, while “Increase of the maximum funding amount per project” (3.5 ± 0.16) and “Increased use of cross-cutting or joint calls” (3.8 ± 0.18) were considered the least important needed changes.

However, when asked “Do you think that the current funding programmes to which you apply to, are rightly designed to support agri-food transformation through agroecology or do you think that some changes are necessary to strength and accelerate this transformation in Europe?” only 54% of respondents answered “No, some changes are necessary”. Specifically, they mainly required: a) greater duration of the projects with a better integration of social aspects together with the other components (quotes 5–6, Table 4); b) more topics expressly designed for agroecology (quotes 7–10 *);* c) more attention to producers, especially smallholder farmers, and to the different actors of the food system (quotes 11–13); d) less bureaucracy, flexibility and change in funding amount and reporting methods (quotes 14–20).

## Discussions

### Key elements of agroecology research

Agroecology embraces several disciplines stemming from agronomy, ecology, sociology, and economics ( Wezel & Soldat, 2009) as also highlighted by our identified research projects which simultaneously address different sustainability dimensions. The combination of these disciplines to produce knowledge through an interdisciplinary approach where researchers collaborate with higher level of integration of goals and concepts ( Mauser *et al.*, 2013) was implemented and preferred with a multidisciplinary approach where scientists collaborate but maintain their disciplinary perspectives. The co-creation of knowledge relevant for tangible problem solving, through the collaboration of researchers from different disciplines and non-academic actors is instead defined as transdisciplinarity ( Mauser *et al.*, 2013; Popa *et al.*, 2015). It is also seen as an interdisciplinarity with the participation of non-academic actors ( Fernández González *et al.*, 2021). Hence, interaction processes with non-academic actors are an important element of transdisciplinary research. This type of research seems to be better implemented by European projects given the greater number of the involved actors and the high degrees of actor interaction achieved in the three phases of the research.

Living labs are increasingly gaining ground as an approach to be used in research projects to strengthen transdisciplinarity and innovation ( McPhee *et al.*, 2021). However, according to our survey with project coordinators, transdisciplinarity in LLs is sometimes reduced to a “buzzword” and the mere involvement of non-academic actors is reduced to a consulting or informative process rather than a deep integration of knowledge because co-innovation and co-knowledge processes are not carried in all projects with LLs. RIs were widely used in agroecology research but data remained available after the end of projects for the scientific community in some of the cases. Indeed, whereas researchers are incentivized to promote open access in the case of scholarly publications because their outcomes become more widely known, there are still some reticence and obstacles for research data sharing and reuse ( Stuart *et al.*, 2018).

Considering the current role of agroecology research in supporting the agri-food transformation in Europe, both surveys suggest strengthening the issues related to redesign (level three) and social, governance aspects and full food systems transformation (level five), given that they obtained the lowest rates of responses. A greater involvement of upstream and downstream value chain actors could further strengthen the food system approach. In general, although we are aware that our study might not represent the whole picture of agroecology research in Europe due to a limited number of answers, it highlights a predominant trajectory more prone to the transformation of the agri-food system rather than its mere incremental change. In fact, according to the percentages of the ACT categories, in both surveys researchers implementing an approach of re-design of the farm/agroecosystem and the whole agri-food system represented the majority.

### Agroecology research and debates

It is important to note that our study has some limitations related to the narrow number of answers. Furthermore, the results of the survey open to all researchers were based only on the responses of individuals from a limited number of European countries, about half of which from Italy. Despite this bias, the comparison of the answers of the two surveys (project coordinators and researchers) also in the conflicting and debated issues related to agroecology resulted consistent. Indeed, our surveys were suitable to capture insights on key aspects of the discussion on agroecology research. An important debate that animates the agroecology scientific community concerns the agricultural model to be considered in research on agroecology ( Buttel, 2003; Levidow *et al.*, 2014). Many researchers see a risk in ensuring support to mainstream forms of agriculture with the implicit possibility of being co-opted by the dominant regime. Considering our identified agroecology research projects, both organic and conventional production systems are addressed. This also seems to be confirmed by the survey open to all agroecology researchers, where organic agriculture, although relevant, does not seem so closely associated with agroecology. However, looking at the percentages of projects in which the use of synthetic inputs is allowed, it seems that these do not cover all projects in which the conventional system is present, especially for EU projects. Therefore, part of the research projects in agroecology that also operate under the conventional farming system seem to push it to shift to organic (in relation to inputs).

Tensions also occur between sustainable intensification and ecological intensification, the former more linked to conventional agriculture with an emphasis on increasing yields without adverse environmental impacts and without the conversion of additional non-agricultural land, the latter aimed at transforming the agricultural system through a knowledge-intensive process that requires optimal management of nature’s ecological functions and biodiversity ( Levidow *et al.*, 2014). In both surveys, ecological intensification was selected and preferred to sustainable intensification thus denoting a predominance of respondents who implement a transformative approach in agroecology research.

Another very heated debate is around whether and to what extent genetic engineering and agroecology are compatible ( Bonny, 2017; Giller *et al.*, 2017; Lotz *et al.*, 2020). These two different visions still coexist. There are researchers favourable to genetically modified organisms when applied to making crops less vulnerable to pests and diseases, whilst others are concerned as they see GMOs as closely connected to interests innervating the so-called productivist model and the related risk of privatizing research results and increasing farmers’ dependence on seeds controlled by multinationals. Differently, the results of our survey on the identified projects show that this double vision on GMOs is inexistent, as only selective breeding and crossbreeding techniques seem to be accepted and implemented in the agroecological research.

### Connections and collaborations in research projects

The SNA showed low values of diameter and mean distance denoting a good connection and collaboration between all the identified 36 countries of the network, although about 22% of the interactions represent occasional partnerships between two countries. France, Italy, Spain, Germany, and United Kingdom show to be most active countries in agroecology research in Europe based on having coordinated many projects. Moreover, these countries, together with the Netherlands and Portugal, often have central stage in the network, showing a good performance in degree centrality. In fact, according to this measure, the nodes in the centre are the ones with the largest number of direct connections with other nodes in the network. Degree centrality is interpreted as an indicator of a country’s activity in agroecology research, its interests and engagement in agroecology research projects, its attractiveness as a partner and readiness for new partnerships ( Divjak *et al.*, 2010). On the contrary, peripherical countries in the network with a low degree of centrality ( *e.g*. Ukraine, Moldavia, Malta) have a low occurrence as partners in agroecology research projects and are the least attractive. However, some peripherical countries in the SNA, such as Ukraine, show a good performance in closeness centrality. Closeness represents a measure of how long it will take to spread information to all other nodes sequentially. Therefore, closeness centrality is a good indicator of the speed of establishing connections, diffusion of innovations and information, and partnership establishment with all involved countries in the network. Time often plays a major role in the process of finding partners and high closeness centrality indicates that a country is well-connected with other countries and can therefore provide partnership in rather short time ( Divjak *et al.*, 2010). Moreover, this measure takes into account both direct and indirect connections. Information can often travel faster through the indirect connections than the direct ones because weights can shorten the path ( Newman, 2001). Although Ukraine is one of the countries with fewer direct connections with other nodes, nevertheless having established strong connections with well-connected countries such as Spain and Italy, it can use these indirect connections to reach all countries of the network more quickly.

Three different groups of countries that show having more partnerships among themselves in agroecology research projects than with other countries were highlighted by our analysis. In general, excluding some exceptions, countries seem to cluster according to the biogeographic regions identified by the European Environment Agency. This is reasonable because it allows partners of agroecology research projects to identify common problems and potential solutions which can be more easily shared and implemented among similar environments. In any case, given the not-so-high value of the maximum modularity, the greater collaboration between countries within each group exists but it is not so strong and the countries of each group collaborate with the countries of the other groups as well. This aspect could be promoted and guaranteed by the funding research programmes themselves, which often require the formation of partnerships within a project that embrace different European areas.

### Policy recommendations

Even if in the past agroecology was not explicitly mentioned in any research funding programmes, now it finally appears in a clear and evident way within both the Horizon Europe framework (2021-2027) and the new partnership “ Accelerating farming systems transition: agroecology living labs and research infrastructures”. Once more fully integrated in these research programmes, funding needs to be properly designed to ensure it promotes the transformative paradigm of agroecology in Europe.

The results of the social network analysis might be exploited by the leaders of international programmes to promote cooperation and spread information and innovation in agroecology more quickly within the European region. For example, peripherical countries and research groups should be supported in connecting to more consolidated networks and active countries, strengthening cooperation to amplify agroecology research at larger European scale. This is especially true for transnational programming which, by its mission, tends to align the different national strategies and support research activities under an agreed vision in order to overcome fragmentation of public research efforts.

In order to support the transformative paradigm of agroecology, topics addressing at least the level three and up to five of Gliessman’s framework should be promoted by research funding programmes. Specifically, those issues that have currently been slightly addressed by research in agroecology (according to our surveys: resilience in level three, social and governance aspects of level four and five) should be given a more prominent attention. To address the social challenges posed by agroecology (especially those related to level four and five), research should involve a greater number of actors from the entire agri-food system, in particular those who are currently less represented such as upstream and downstream value chain actors.

New ways of knowledge production based on the involvement and interaction of non-academic actors from the entire agri-food system into the research process are fundamental to increase the impact of the research, enhancing territoriality and control at the local level. Specifically, a transdisciplinary research environment should be strengthened and emphasized by research funding programmes to address serious societal challenges on the ground ( Schneider *et al.*, 2019).

Funding research programmes in agroecology must support and require transdisciplinary research more effectively, explicitly demanding transdisciplinary designs and processes. In this respect, the new partnership on agroecology is moving in the right direction having chosen LLs as a strong point to accelerate and strengthen co-innovation. However, as highlighted by the results of our surveys, further efforts must be made to identify those elements that a LL must have in order to truly guarantee the implementation of transdisciplinary approaches thus allowing a more rapid diffusion of agroecological innovation. At the same time, designers of the funding programmes must be aware that transdisciplinary efforts imply a much-needed flexibility. Indeed, ongoing interactions with actors often require some adaptations of the original research proposal. Institutional and procedural innovation to introduce further flexibility in budget management and project partnership redefinition is advisable. Funding programmes should encourage researchers’ willingness in data sharing and reuse to fortify knowledge in agroecological processes by promoting proper rewards and requiring mandatory data sharing agreements in the research projects.

Our results also bring forward the need to increase the duration of projects dealing with agroecology, as contributions to societal transformation often require more time to unfold. Although not being a primary concern of the respondents to our surveys, an increase of agroecology project complexity in terms of duration, number and type of actors involved, better inclusion of social and governance aspects together with the environmental and economic dimensions, will have an impact on budget dimension. This should be taken into consideration especially by the future transnational research planning, that should be designed in order to promote the funding of properly sized projects, thus avoiding small projects which might result too simplified as many of the transnationally financed ones in the past, as well as very large not-efficiently manageable ones. On the basis of the above reported considerations, some policy recommendations to foster the transformative role of agroecology research in Europe can be formulated ( Table 5).

**Table 5.** Policy recommendations to foster the transformative role of agroecology research in Europe.

1.	Establish research programmes that consider the entire agri-food system and its actors, not only the agronomic field and farming scales.
2.	Strengthen research cooperation and networks at European scale by lowering the barriers that hinders the connection and participation of the currently less involved countries.
3.	Promote research programmes addressing, at least, the level 3 of Gliessman′s framework and going beyond, including social and governance aspects of level 4 and 5. On the other hand, diminish research programmes addressing only the level 1 and 2.
4.	Design research programmes that strengthen transdisciplinary research, and explicitly demand the implementation of transdisciplinary designs and processes.
5.	Enhance the involvement of a greater number of actors from the entire agri-food system, in particular those who have been less represented thus far, such as upstream and downstream value chain actors, and the non-economic actors of the food system (i.e., citizens).
6.	Identify important elements and traits of the Agroecology Living Labs to truly guarantee the implementation of transdisciplinary approaches
7.	Promote appropriate policies regarding scientific data to guarantee data sharing and reuse within the scientific community (i.e., rewards, mandatory data sharing agreements)
8.	Introduce institutional and procedural innovation to guarantee higher flexibility in the implementation of research projects, especially within budget and partnership management
9.	Increase the duration of projects that deal with agroecology
10.	Frame research programmes in a way that does not allow small projects whose results might be too simplified, as well as very large one that cannot be efficiently managed

## Conclusions

Our study shows a great dynamic and already existing European research network on agroecology with a predominant trajectory more prone to the transformation of the agri-food system rather than its mere incremental change. Nevertheless, today more than ever, given that the boundaries between the productivist and the transformative models are becoming blurred, it is necessary to ensure that the transformative role of agroecology be made more decipherable and visible. Mainly, our outcomes suggest fostering the transformative role of research in agroecology by considering the whole agri-food system together with its various actors, reducing research issues related prevalently to the system incremental change, strengthening transdisciplinary research, increasing complexity, budget, and project duration, and amplifying agroecology research and cooperation at larger European scale.

Given the new calls and the partnership within the new Horizon Europe framework which are being launched and designed explicitly for agroecology, both European and transnational funding research programmes may now more easily promote the transformative paradigm. This may lead to driving and harmonizing the national funding research programmes for agroecology of the various European countries towards this vision, too. Accordingly, the findings of our study have the ambition to contribute achieving this vision by providing science-based recommendations useful to steering research policy makers’ actions.

## Ethics and consent

No relevant ethical issues were identified by AE4EU project regarding studies with humans and human interventions. For the collection of personal data, detailed information on the procedures for data collection, storage, protection, retention, and destruction, and confirmation that they comply with national and EU legislation are described in the deliverable D8.2 submitted in March 31, 2021, and accepted by the EU Commission. The project Ethics committee, consisting of the project coordinator, the data protection and management officer, as well as representative of selected partners of the Ethics work package validated the questionnaires used in the surveys of this study. Written informed consent was also obtained from participants to the surveys to use their answers and quotations for research and publication

## Data Availability

Zenodo: Database on Projects, Programmes, and Institutions (PPIs) related to agroecology. DOI: https://doi.org/10.5281/zenodo.7248937 This project contains the following underlying data: AE4EU_db_PPIs_public_version.xlsx. This database holds the list of projects, funding programmes, and institutions (PPIs) dealing with agroecology research in Europe identified in the mapping activities carried out in the Task 1.3 of AE4EU project ( https://www.ae4eu.eu/). The lists of the projects and funding programmes identified at European and the transnational levels were used in this paper Zenodo: Social Network analysis on European countries involved in agroecology research. DOI: https://doi.org/10.5281/zenodo.7253382 This project contains the following underlying data: SNA_on_agroecology_research_in_Europe.xlsx. This dataset contains data related to the social network analysis performed including European countries involved in agroecology research projects. It consists of two sheets: 1. *Indexes* where values of some measures for each identified country in the social network analysis are reported; *Edge_weights* where the weights for each edge between two countries are provided according to the times two countries cooperated together for a project. Zenodo: Survey for coordinators of agroecology research projects. DOI: https://doi.org/10.5281/zenodo.7254217 This project contains the following underlying data: Project_coordinators_survey.csv. This dataset contains data related to the survey launched within AE4EU project for the coordinators of agroecology research projects funded by European, transnational, and national programmes identified in the mapping activities. Responses from coordinators of European and the transnational funding programmes were only used in this paper. Zenodo: Survey for researchers involved in Agroecology. DOI: https://doi.org/10.5281/zenodo.7254287 This project contains the following underlying data: Agroecology_Researchers_survey.csv. This dataset contains data related to the survey launched within AE4EU project for the researchers involved in agroecology; Data are available under the terms of the Creative Commons Attribution 4.0 International license (CC-BY 4.0). Zenodo: Survey for coordinators of agroecology research projects. DOI: https://doi.org/10.5281/zenodo.7254217 This project contains the following extended data: Project_coordinators_questionnaire_structure.pdf, where the structure of the questionnaire related to the survey is provided Zenodo: Survey for researchers involved in Agroecology. DOI: https://doi.org/10.5281/zenodo.7254287 This project contains the following underlying data: Agroecology_Researchers_questionnaire_structure.pdf. The structure of the questionnaire related to the survey is provided Data are available under the terms of the Creative Commons Attribution 4.0 International license (CC-BY 4.0).
