# Supplementary material for: Agroecology research in Europe funded by European and transnational programmes: current status and perspectives
Source: Open Res Eur. 2023 Mar 29;2:139. Originally published 2022 Dec 15. [Version 2] doi: 10.12688/openreseurope.15264.2 (PMC10445821; doi:10.12688/openreseurope.15264.2)
Supplement: Peer review history

# Peer review: Reviewer response for version 2

**DOI:** 10.21956/openreseurope.17069.r31079

**Published:** 2023-04-12

**Authors:** Les Levidow

**Affiliations:** 1 Open University, Milton Keynes, UK

I have read the authors' response with excerpts from the revised article. I find them satisfactory: a significant improvement in the article. It will help sharpen analyses of agroecology research agendas and proposals for improvement.

Is the study design appropriate and does the work have academic merit?

Yes

Is the work clearly and accurately presented and does it cite the current literature?

Yes

If applicable, is the statistical analysis and its interpretation appropriate?

Partly

Are all the source data underlying the results available to ensure full reproducibility?

Yes

Are the conclusions drawn adequately supported by the results?

Partly

Are sufficient details of methods and analysis provided to allow replication by others?

Yes

Reviewer Expertise:

Interdisciplinary approaches to the hegemonic agri-food system versus agroecological alternatives; Transdisciplinary methods for Participatory Action Research with practitioners on such issues.

I confirm that I have read this submission and believe that I have an appropriate level of expertise to confirm that it is of an acceptable scientific standard.

# Peer review: Reviewer response for version 2

**DOI:** 10.21956/openreseurope.17069.r31080

**Published:** 2023-04-04

**Authors:** Manuel Gonzales de Molina

**Affiliations:** 1 Agro-ecosystems History Laboratory, Pablo De Olavide University, Seville, Spain

The authors have addressed the suggestions and comments in my peer review report appropriately. I believe that the paper is now ready for indexing.

Is the study design appropriate and does the work have academic merit?

Yes

Is the work clearly and accurately presented and does it cite the current literature?

Yes

If applicable, is the statistical analysis and its interpretation appropriate?

Yes

Are all the source data underlying the results available to ensure full reproducibility?

Yes

Are the conclusions drawn adequately supported by the results?

Yes

Are sufficient details of methods and analysis provided to allow replication by others?

Partly

Reviewer Expertise:

Agroecology, Agricultural History, Ecological Economics

I confirm that I have read this submission and believe that I have an appropriate level of expertise to confirm that it is of an acceptable scientific standard.

# Peer review: Reviewer response for version 1

**DOI:** 10.21956/openreseurope.16506.r30504

**Published:** 2023-02-07

**Authors:** Manuel Gonzales de Molina

**Affiliations:** 1 Agro-ecosystems History Laboratory, Pablo De Olavide University, Seville, Spain

The paper presents the results of a research carried out within a European AE4EA project. I consider this research to be very relevant, just when the European F2F and Biodiversity 2030 strategies are starting to be developed, when some Horizon Europe calls are containing Agroecology as a topics and, in particular, when the future Agroecological Living Labs partnership is being drawn up. The results partially reflect the state of research being done in Europe, its virtues and its shortcomings. Overall both the methodological approach and the research development, including the results presentation are correct. I have little to object to. However, I would like to highlight some of its conclusions, which seem to me to be particularly important, and to make some comments that may improve the text, if the authors consider it appropriate.

*General comments and suggestions*

 1. The title should be changed as the conclusions and the study refer to H2020 and ERA-Net projects and not to European research as a whole. Throughout the text it is clear that the paper does not refer to all agroecological research carried out in the EU and UK countries; but this necessary clarification should be made at the very beginning of the paper. The title implies that the paper refers to European research in agroecology; but the paper refers to only a very small part of all the agroecological research that is carried out with the support of national and regional research agencies of the member states and by private foundations and associations, and even by companies in the sector.

 Indeed, on page 3 it is explicitly stated that "the main objective of this study was to map research projects related to agroecology in Europe with the aim to". However, this statement suggests that all projects and all research in Europe, whatever their scope and whatever the national, regional or private funding agency, have been included in the research. The paper refers only to H2020 research projects and transnational projects included in partnerships, i.e. co-funded by several countries. Given the specificity of this type of call, the results of this research must be handled with great care when attempting to generalise them to all research on agroecology in Europe.

 It should be noted from the beginning that: i) national projects are not included; ii) doctoral theses are not included (there are databases available for consultation); iii) the closed and exclusive method of calling for H2020 projects and the absence until a few years ago of topics in agroecology make less representative the research presented in this paper; iv) the participatory and local nature of agroecology research means that many research projects are carried out outside of European programmes and therefore this research is only representative of the H2020 and some partnerships projects on agroecology.

 2. The paper often refers to countries and organizations such as FAO supporting agroecology. But it should be clear that some governments have supported or support the development of agroecology and that this does not mean that they are forerunners of agroecology in Europe. A distinction should be made between governments --and their action in the field of agroecological research—and researchers and movements and their role in the development of local or regional agroecological experiences. In this sense, it is a bit confusing to say that "Under the initiative of Food and Agriculture Organisation (FAO), France, Italy, Hungary, and Switzerland joined the so-called group of the Friends of Agroecology, manifesting an explicit intention to support international efforts in this direction (Bruil *et al.*, 2019)…These countries, acting as agroecological forerunners at European level, might have gained a leading position in promoting agroecology, imprinting different visions and towing the European research and innovation communities”. But the effective support of the Swiss or Hungarian governments for agroecology can be seriously questioned, as well as the effort currently being invested by France and Italy from a budgetary point of view: In any case, it should be clarified that the paper refers to research that is carried out at European level and from the main European calls.

 3. A central place in the paper is given to those European projects that have a clear "transformative" character of the food system in Europe. I agree with the relevance of this criterion in selecting which projects are truly agroecological and which ones actually use the word but in an opportunistic way. However, it would be good if the authors included in the text a more precise definition of the term transformative, given its relevance to the co-opted use of agroecology, and given the justified relevance that the authors give to this concept. It is supposed to change the food system from its current configuration and promote a more sustainable one. It is difficult to provide a detailed definition of this in a paper devoted to a different topic, but at least some basic features should be given to clarify what we mean by transforming the food system, from production to consumption.

 4. On page 3 it says: “In contrast to this transformative agenda, agroecology has been adopted by actors who promote conventional agriculture and the agro-industrial productivist model (Holt- Gimenez & Altieri, 2013), through conservation agriculture and sustain able intensification approaches geared towards the increase of productivity”.

 I think it would be useful to qualify this statement and show that in fact agroecology has been "adapted" and not “adopted” by actors who promote conventional agriculture and the agro-industrial productivist model. Indeed, this adaptation has meant the promotion of a weakened version of agroecology, stripping it of its transformative character and only adopting those principles of agroecology that are least opposed to the continuity of the industrial productivist model and, therefore, best suited to its interests.

 5. I advise a careful revision of the references because some authors' names are misspelled. For example: "Industrial agriculture is questionable (Alonso-Fradeyas *et al*., 2020)". It should be written "Fradejas".

*Methodological section*

 1. The key words used "to include more projects that did not explicitly mention agroecology but that could be referred to agroecologically linked approaches and systems" are listed on page 4. The authors have considered some additional key words such as: agroforestry, silvopasture, silvoarable, food justice, food system, territorial food system, food sovereignty and rural development. However, it is not clear what criteria have been taken into account to consider that these very general lists correspond to projects with agroecological content. Moreover, the key words selected are so few that it is possible that other sustainable agriculture projects that may be agroecological have been left out. I miss, for example, key words of an agronomic nature, nitrogen fluxes, GHG emissions, energy, etc...

 2. I find it very striking that only "three projects were instead obtained from Organic e-prints after setting the filters relating to the years (2014 or later) and the English language". Why is this? Is it the type of projects or the e-print format that explains why there are only three? It would be worth venturing an explanation...

 3. It would be good if the authors could provide a clearer and less succinct explanation of why they used a Social Network Analysis (SNA) and why this is the most appropriate method for this type of research.

 4. The two surveys conducted among PIs and researchers seem very appropriate and provide high quality and clear information on the type of research being carried out at the European level. The results show responses that are in line with what most of us researchers working in the field of agroecology intuitively believe: “a) greater duration of the projects with a better integration of social aspects together with the other components (quotes 5–6, Table 4); b) more topics expressly designed for agroecology (quotes 7–10); c) more attention to producers, especially smallholder farmers, and to the different actors of the food system (quotes 11–13); d) less bureaucracy, flexibility and change in funding amount and reporting methods”. However, the paper should have also described or asked about the barriers to entry of agroecological research into the European public funding system. For example, what percentage of the total amount of funding is allocated, and why are there so few of them?

*On the results:*

 1. Although they cannot be considered as more than representative of the projects studied at European level (H2020, P2Ps, etc.), the results are very interesting. Of particular importance is the finding that: “Living labs are increasingly gaining ground as an approach to be used in research projects to strengthen transdisciplinarity and innovation (McPhee *et al.,* 2021). However, according to our survey with project coordinators, transdisciplinarity in LLs is sometimes reduced to a “buzzword” and the mere involvement of non-academic actors is reduced to a consulting or informative process rather than a deep integration of knowledge because co-innovation and co-knowledge processes are not carried in all projects with LLs”. This feature contrasts with the firmly established tradition in agroecological research of using participatory action research, which is now beginning to adopt the format of LLs and co-creation of knowledge. The participatory action research tradition has been the most frequent way for developing agroecological research in Latin America and some European countries since the 1990s, often with little or no connection to the academic world that has the capacity to lead and obtain European projects.

 2. The main conclusion that the authors draw from their research is promising: “In general…it highlights a predominant trajectory more prone to the transformation of the agri-food system rather than its mere incremental change. In fact, according to the percentages of the ACT categories, in both surveys researchers implementing an approach of re-design of the farm/agroecosystem and the whole agri-food system represented the majority”. But it should also be noted that in general the number of projects remains low and that funding is also scarce. One might even ask whether "more projects with smaller budgets" is not a better way of increasing research and the number of researchers than the current European project scheme.

Is the study design appropriate and does the work have academic merit?

Yes

Is the work clearly and accurately presented and does it cite the current literature?

Yes

If applicable, is the statistical analysis and its interpretation appropriate?

Yes

Are all the source data underlying the results available to ensure full reproducibility?

Yes

Are the conclusions drawn adequately supported by the results?

Yes

Are sufficient details of methods and analysis provided to allow replication by others?

Partly

Reviewer Expertise:

Agroecology, Agricultural History, Ecological Economics

I confirm that I have read this submission and believe that I have an appropriate level of expertise to confirm that it is of an acceptable scientific standard, however I have significant reservations, as outlined above.

# Response

**Published:** 2023-03-20

**Authors:** Ileana Iocola

**Affiliations:** Council for Agricultural Research and Economics, Research Centre for Agriculture and Environment (CREA-AA), Italy

We thank the referee for the careful review which helped improve the manuscript. Please find below a detailed point-by-point response to all comments.

**The paper presents the results of a research carried out within a European AE4EA project. I consider this research to be very relevant, just when the European F2F and Biodiversity 2030 strategies are starting to be developed, when some Horizon Europe calls are containing Agroecology as a topics and, in particular, when the future Agroecological Living Labs partnership is being drawn up. The results partially reflect the state of research being done in Europe, its virtues and its shortcomings. Overall both the methodological approach and the research development, including the results presentation are correct. I have little to object to. However, I would like to highlight some of its conclusions, which seem to me to be particularly important, and to make some comments that may improve the text, if the authors consider it appropriate.**

 R: We thank the reviewer for these considerations. We agree with him that the work only partially reflects the state of the research in Europe (see our next answer - R1 under General comments and suggestion). In fact, in this work our aim was to provide support in the design of the research funding programmes related to agroecology framed within the Horizon framework. For this reason, we analysed specifically research projects related to this framework.

**General comments and suggestions (S)**

**Q1-S)** **The title should be changed as the conclusions and the study refer to H2020 and ERA-Net projects and not to European research as a whole. Throughout the text it is clear that the paper does not refer to all agroecological research carried out in the EU and UK countries; but this necessary clarification should be made at the very beginning of the paper. The title implies that the paper refers to European research in agroecology; but the paper refers to only a very small part of all the agroecological research that is carried out with the support of national and regional research agencies of the member states and by private foundations and associations, and even by companies in the sector. Indeed, on page 3 it is explicitly stated that "the main objective of this study was to map research projects related to agroecology in Europe with the aim to". However, this statement suggests that all projects and all research in Europe, whatever their scope and whatever the national, regional or private funding agency, have been included in the research. The paper refers only to H2020 research projects and transnational projects included in partnerships, i.e. co-funded by several countries. Given the specificity of this type of call, the results of this research must be handled with great care when attempting to generalise them to all research on agroecology in Europe. It should be noted from the beginning that: i) national projects are not included; ii) doctoral theses are not included (there are databases available for consultation); iii) the closed and exclusive method of calling for H2020 projects and the absence until a few years ago of topics in agroecology make less representative the research presented in this paper; iv) the participatory and local nature of agroecology research means that many research projects are carried out outside of European programmes and therefore this research is only representative of the H2020 and some partnerships projects on agroecology.**

 R1-S: We agree with the reviewer. In fact, our preliminary idea was to include the national level in the mapping activities, too. As it was difficult to find information in English language on national research projects in the different European countries, we have directly contacted by email several national funding agencies in order to obtain these data. AE4EU partners also supported this search at national level providing additional information. At the end, we identified 300 national projects covering 15 Countries. Unfortunately, we received only 32 replies for the coordinators of these research projects belonging to nine different countries. These replies were therefore too few to justify a country-level analysis. For this reason, we decided to report data at national level only in the project deliverable, while in this paper we preferred focusing our analysis on the research projects at European and transnational level. In order to clarify this aspect, we have reformulated the aims of our work delineating the area of our analysis. They are the following:

 “ *With the main objective to support the design of funding research programmes within the Horizon Europe framework and the new partnership on agroecology, this study mapped research projects related to agroecology funded by the European Union and/or co-funded by Member States to: i) characterize this research in terms of implementation of agroecology elements and evaluate if this current agroecology research really contributes to create transformative alternatives to the current agri-food regime; ii) understand connections among European countries participating to this research and identify the most powerful and influential countries investing in agroecology research and the less active ones, whose involvement should instead be promoted; and iii) provide recommendations for future research agendas within these funding research programmes to better strengthen agroecology and its transformative role in Europe*”. Moreover, we have replaced the previous title “ *Agroecology research in Europe: current status and perspectives*” with “ *Agroecology research in Europe funded by European and transnational programmes: current status and perspectives*”. 

**Q2-S)** **The paper often refers to countries and organizations such as FAO supporting agroecology. But it should be clear that some governments have supported or support the development of agroecology and that this does not mean that they are forerunners of agroecology in Europe. A distinction should be made between governments --and their action in the field of agroecological research—and researchers and movements and their role in the development of local or regional agroecological experiences. In this sense, it is a bit confusing to say that "Under the initiative of Food and Agriculture Organisation (FAO), France, Italy, Hungary, and Switzerland joined the so-called group of the Friends of Agroecology, manifesting an explicit intention to support international efforts in this direction (Bruil *et al.*, 2019)…These countries, acting as agroecological forerunners at European level, might have gained a leading position in promoting agroecology, imprinting different visions and towing the European research and innovation communities”. But the effective support of the Swiss or Hungarian governments for agroecology can be seriously questioned, as well as the effort currently being invested by France and Italy from a budgetary point of view: In any case, it should be clarified that the paper refers to research that is carried out at European level and from the main European calls.**

 R2-S: These sentences have been inserted to highlight that there is a strong attention to agroecology in recent years. In fact, some governments have decided to support the development of agroecology at minor or greater level. In order to consider the concerns highlighted by the reviewer and to distinguish the actions carried out by governments and researchers, we have inserted the term “governments” in the sentence which is now the following “ *This interest in agroecology did not emerge simultaneously and with similar intensity in the different European territories and countries. Under the initiative of Food and Agriculture Organisation (FAO), the governments of France, Italy, Hungary, and Switzerland joined the so-called group of the Friends of Agroecology, manifesting an explicit intention to support international efforts in this direction (Bruil et al., 2019)*”. Moreover, we decided to remove the following sentence from the manuscript: “ *These countries, acting as agroecological forerunners at European level, might have gained a leading position in promoting agroecology, imprinting different visions and towing the European research and innovation communities*”. Lastly, the paper was also modified to better highlight that it is referred to the research carried out at European and transnational level (see R1-S)

**Q3-S)** **A central place in the paper is given to those European projects that have a clear "transformative" character of the food system in Europe. I agree with the relevance of this criterion in selecting which projects are truly agroecological and which ones actually use the word but in an opportunistic way. However, it would be good if the authors included in the text a more precise definition of the term transformative, given its relevance to the co-opted use of agroecology, and given the justified relevance that the authors give to this concept. It is supposed to change the food system from its current configuration and promote a more sustainable one. It is difficult to provide a detailed definition of this in a paper devoted to a different topic, but at least some basic features should be given to clarify what we mean by transforming the food system, from production to consumption.**

 R3-S: In order to better outline what we intend with the “transformative” approach, we added some sentences related to the Gliessman’ framework on the food system changes in the Introduction. In the updated version of the manuscript, after the following sentence: “ *It represents a collective action mode for transforming the dominant agri-food regime and creating alternatives (Levidow et al., 2014) towards a process of redesigning food systems to achieve ecological, and socio-economic sustainability (Gliessman, 2016)*” we added *“Specifically, Gliessman (2014) proposed a theoretical framework based on five levels to outline the process of transformation of agri-food systems through agroecology emphasizing the need to go beyond the farm level solutions to include the interactions within the whole food system, from production to consumption. The first two levels include the incremental changes, with no transformational aims. They mainly focus on the increase of input efficiency and substitution of inputs/practices at the farm level. The transformational changes start from the third level which focuses on a redesign of agroecosystems through diversity, synergies, and strengthening resilience. Levels four and five address the food system changes with the re-connection of consumers to farmers through community, business, and policy support and incentives, and the rebuilding of an equitable, just, participatory, fully sustainable global food system”.*

**Q4-S) On page 3 it says: “In contrast to this transformative agenda, agroecology has been adopted by actors who promote conventional agriculture and the agro-industrial productivist model (Holt- Gimenez & Altieri, 2013), through conservation agriculture and sustain able intensification approaches geared towards the increase of productivity”.**

**I think it would be useful to qualify this statement and show that in fact agroecology has been "adapted" and not “adopted” by actors who promote conventional agriculture and the agro-industrial productivist model. Indeed, this adaptation has meant the promotion of a weakened version of agroecology, stripping it of its transformative character and only adopting those principles of agroecology that are least opposed to the continuity of the industrial productivist model and, therefore, best suited to its interests.**

 R4-S: In order to show that agroecology was “adapted” by actors who promote conventional agriculture and the agro-industrial productivist model, we added the following sentence in the manuscript: “ *Indeed, in this context, the term agroecology was also used by some agri-input supply companies such as Syngenta (Levidow et al., 2014) to promote no till methods for enhancing soil fertility and reducing erosion with the inclusion of herbicide treatments and herbicide-tolerant crops*”.

**Q5-S) I advise a careful revision of the references because some authors' names are misspelled. For example: "Industrial agriculture is questionable (Alonso-Fradeyas *et al*., 2020)". It should be written "Fradejas".**

 R5-S: We apologize for this error. We corrected it and we also checked the rest of the references reported in the manuscript

**Methodological section (M)**

**Q1-M) The key words used "to include more projects that did not explicitly mention agroecology but that could be referred to agroecologically linked approaches and systems" are listed on page 4. The authors have considered some additional key words such as: agroforestry, silvopasture, silvoarable, food justice, food system, territorial food system, food sovereignty and rural development. However, it is not clear what criteria have been taken into account to consider that these very general lists correspond to projects with agroecological content. Moreover, the key words selected are so few that it is possible that other sustainable agriculture projects that may be agroecological have been left out. I miss, for example, key words of an agronomic nature, nitrogen fluxes, GHG emissions, energy, etc...**

 R1-M: This was a critical issue. We are aware that the keywords we selected are not able to cover all issues of agroecology, but they address the most relevant ones. Keywords such as nitrogen fluxes, GHG emissions, and energy could be related to agroecology (especially considering the evaluation of the impacts/effects arisen from the implementation of system changes). Anyway we think these keywords are more referred to sustainability of agri-food systems. Maybe we missed some keyworks referred to crop diversification or agrobiodiversity but we realized that we obtained many projects related to these issues (i.e., DIVERIMPACTS, DiverFarming, DIVERSIFOOD, etc.) with the keyword “agro-ecolog*”. Moreover, for the transnational projects, it was not possible to set any keywords so we checked all the available projects. Lastly, as reported in the paper, the list of the projects was shared and discussed with partners of AE4EU project which also added some additional projects not covered by our desktop research.

**Q2-M) I find it very striking that only "three projects were instead obtained from Organic e-prints after setting the filters relating to the years (2014 or later) and the English language". Why is this? Is it the type of projects or the e-print format that explains why there are only three? It would be worth venturing an explanation...**

 R2-M: We discussed this issue directly with people involved in the management of Organic e-prints database. Organic e-print is a very useful and powerful open access archive as it allows to select and define many options in the search criteria. The only aspect that we believe needs improvement is that of stimulating researchers to fill and update the archive, especially for projects. Indeed, what we noticed using this useful archive in our work on researching projects related to agroecology is that not all projects appear once the advanced search criteria have been set, especially if we set up a query considering only “Project description” in the EPrint type. On the contrary, if we set up any EPrint type keeping the same keyword, the results increase considerably because also papers, proceedings, thesis, etc. appear. So, our suggestion is to stimulate the coordinators of the organic projects that have been funded to update the archive also for the Eprint type related to the “project description”, not only for paper, abstract, etc. In any case, overall, in our desktop research we were able to retrieve many of the transnational projects because we also consulted other database (i.e., ERA-LEARN database) where the same information is reported.

**Q3-M) It would be good if the authors could provide a clearer and less succinct explanation of why they used a Social Network Analysis (SNA) and why this is the most appropriate method for this type of research.**

 R3-M; We have expanded the introduction adding these following sentences: “ *Agroecology is also increasingly gaining attention in the context of funding research programmes as it is now explicitly mentioned in a clear and evident way within both the Horizon Europe framework (2021-2027) and the new partnership “Accelerating farming systems transition: agroecology living labs and research infrastructures”. Once more fully integrated in these research programmes, funding needs to be properly designed to ensure it promotes the transformative paradigm of agroecology across Europe. With the aim to align the different countries under this common transformative vision, participation and access to this funding should be guaranteed and promoted by the research programmes to all countries especially those less involved in agroecology or having different agricultural goals or interests. Previous studies based on network theory (Enger and Gulbrandsen, 2020; Protogerou et al., 2013) reported that few countries access to the projects and related resources within European Framework programmes. An investigation of the degree of involvement of the different countries in European research projects related to agroecology as well as their networking and relations can help to highlight useful insights for the funders’ perspective*”. Moreover, we reformulated the second aim of our work related to SNA in the following: “ *understand connections among European countries participating to this research and identify the most powerful and influential countries investing in agroecology research and the less active ones whose involvement should instead be promoted*”

**Q4-M)** **The two surveys conducted among PIs and researchers seem very appropriate and provide high quality and clear information on the type of research being carried out at the European level. The results show responses that are in line with what most of us researchers working in the field of agroecology intuitively believe: “a) greater duration of the projects with a better integration of social aspects together with the other components (quotes 5–6, Table 4); b) more topics expressly designed for agroecology (quotes 7–10); c) more attention to producers, especially smallholder farmers, and to the different actors of the food system (quotes 11–13); d) less bureaucracy, flexibility and change in funding amount and reporting methods”. However, the paper should have also described or asked about the barriers to entry of agroecological research into the European public funding system. For example, what percentage of the total amount of funding is allocated, and why are there so few of them?**

 R4-M: Funding allocation to agroecology is a very relevant issue. However, this aspect is out of the scope of our work that was carried out within AE4EU project. Indeed, the analysis of the existing (public and private) funding schemes for agroecology across Europe and the design of future schemes are the aim of another work carried out within our same project (please, see https://www.ae4eu.eu/agroecology-in-europe/funding/).

**On the results (R)**

**Q1-R)** **Although they cannot be considered as more than representative of the projects studied at European level (H2020, P2Ps, etc.), the results are very interesting. Of particular importance is the finding that: “Living labs are increasingly gaining ground as an approach to be used in research projects to strengthen transdisciplinarity and innovation (McPhee *et al.,* 2021). However, according to our survey with project coordinators, transdisciplinarity in LLs is sometimes reduced to a “buzzword” and the mere involvement of non-academic actors is reduced to a consulting or informative process rather than a deep integration of knowledge because co-innovation and co-knowledge processes are not carried in all projects with LLs”. This feature contrasts with the firmly established tradition in agroecological research of using participatory action research, which is now beginning to adopt the format of LLs and co-creation of knowledge. The participatory action research tradition has been the most frequent way for developing agroecological research in Latin America and some European countries since the 1990s, often with little or no connection to the academic world that has the capacity to lead and obtain European projects.**

 R1-R: We strongly agree with the reviewer. Often, in European research related to agroecology, researchers have highlighted problems carrying out a truly transdisciplinary approach because it takes time and researchers have to deal with stringent deadlines both during the preparation of the proposal and in its implementation. We believe that an increase of the duration of projects related to agroecology can contribute to overcome this issue.

**Q2-R)** **The main conclusion that the authors draw from their research is promising: “In general…it highlights a predominant trajectory more prone to the transformation of the agri-food system rather than its mere incremental change. In fact, according to the percentages of the ACT categories, in both surveys researchers implementing an approach of re-design of the farm/agroecosystem and the whole agri-food system represented the majority”. But it should also be noted that in general the number of projects remains low and that funding is also scarce. One might even ask whether "more projects with smaller budgets" is not a better way of increasing research and the number of researchers than the current European project scheme.**

 R2-R: We reported here the same answer (R6) provided to the other reviewer who addressed a comment on the same issue: The promotion of small projects is also considered a way to more effectively and closely involve a wider audience of actors not necessarily or poorly familiar with the administrative procedures requested by the research funding procedures, the administrative burdens in assembling large consortia and the difficulties of managing big projects. Also, in low sized projects, territorial and conceptual boundaries can be more sharply definable, enhancing the peer participation of different actors, increasing the co-creation potential and mitigating the risk of not-properly tailored research actions. However, we also recorded tensions in favour of complexity, interpreted as a way to avoid over-simplification of processes and shallow interactions that the straightforward and linear approaches might imply. In our opinion, these two lines of thinking are not divergent and should be simultaneously considered. We highlighted the outcomes obtained in this regard in our recommendations to advice the research programme planners to consider the issue of project size not only in relation to the budget available, but also considering the project topic(s), the scale of intervention and the expected impact. In the revised version, we expanded the recommendation #10 to be more explicit and possibly exhaustive; now the text is the following:

 “ *Frame research programmes in a way that does not allow small projects whose results might be too simplified, as well as very large one that cannot be efficiently managed. Envision projects dimension also considering the topic(s), the scale of intervention and the expected impact*."

# Peer review: Reviewer response for version 1

**DOI:** 10.21956/openreseurope.16506.r30502

**Published:** 2023-01-24

**Authors:** Les Levidow

**Affiliations:** 1 Open University, Milton Keynes, UK

This paper has tensions among different emphases, both analytically and normatively. The Abstract speaks about agroecology for ‘more sustainable agriculture and food systems’, vaguely defined. More specifically, it mentions ‘a predominant trajectory of agroecology research prone to the transformation of the agri-food system’, at least as an aim. Elsewhere the paper distinguishes between transformational versus merely incremental changes, corresponding to the upper versus lower levels of Gliessman’s schema, respectively. That distinction potentially corresponds to interdisciplinary versus transdisciplinary research, the latter having greater involvement of non-production actors across the supply chain, especially value-chain actors. Although plausible, such correspondences ideally should be more sharply analysed in practice, beyond simply projects’ aims.

 Those differences were investigated through a literature review of research projects and a questionnaire-survey of some. As an important finding, ‘Surveys reported a limited participation of value chain actors, while researchers, farmers, and their associations were the most involved’. This practical reality apparently contradicts many projects’ aims corresponding with upper levels of Gliessman’s schema, which emphasises short food-supply chains of a solidarity economy. The latter terms are absent from the analysis of projects, for unclear reasons: because they are absent from all the projects?  It would be helpful to know what extent or type of downstream actors were involved, on the one hand, and what limited their involvement, on the other hand.

 In surveying EU research projects, the paper classifies them along the above lines by assigning percentages. EU projects have significant variations but rate more favourably (for upper levels) than international ones. As an important finding, the paper describes difficulties or limitations in implementing transformative aims, even through transdisciplinary research (Table 4). For example, sometimes ‘the mere involvement of non-academic actors is reduced to a consulting or informative process rather than a deep integration of knowledge because co-innovation and co-knowledge processes are not carried in all projects with LLs’ (Living Labs). Hence the statistical analysis can indicate only intentions, not necessarily practices. The paper could usefully identify projects which substantively fulfilled the more ambitious aims (or did not) and analyse how/why.

 As another parameter, the paper does a social network analysis (SNA) of EU projects, identifying actors’ interconnections across projects and countries. The results indicate significant differences among projects. It remains unclear how these relate to the above distinction between incremental versus transformative aims, likewise between interdisciplinary versus transdisciplinary research. So the SNA lacks clear relevance to the overall argument. The paper could clarify whether this is a loose end warranting further analysis and perhaps more information. 

 The Conclusion makes a cryptic comment, ‘the boundaries between the productivist and the transformative models are becoming blurred’. By contrast, the Introduction paragraph 2 implies that there is a clear boundary between different agendas appropriating agroecological techniques. So the ‘blurred boundaries’ remain unclear — in their own right, in relation to the empirical results and in relation to research agendas. If true, then it might complicate the paper’s key distinctions, as summarised in my first paragraph. 

 The Conclusion makes several recommendations for how structural changes in EU research funding could better facilitate research for a transformational agroecology. Yes, certainly ‘strengthening transdisciplinary research and increasing complexity’, especially in expecting greater involvement by value-chain actors. However, larger budgets and longer duration would impose greater administrative burdens in assembling consortia and managing projects, thus precluding or marginalising potential partnerships that may have important contributions. Indeed, when I briefly represented a pro-agroecology scientists’ consortium in EU research discussions, I had a mandate to advocate more projects with smaller budgets.

 Some recommendations here seem like an administrative fix for more fundamental limitations that need different solutions in transforming research cultures and institutions. In particular, value chains potentially shape production choices, methods and outlets -- but may seem less ‘scientific’ issues for justifying research. This relates to the above lacuna as regards the limited value-chain actors so far involved, even in projects espousing transdisciplinary research. The paper could better reflect on those limitations and means to overcome them for transformative agendas

Is the study design appropriate and does the work have academic merit?

Yes

Is the work clearly and accurately presented and does it cite the current literature?

Yes

If applicable, is the statistical analysis and its interpretation appropriate?

Partly

Are all the source data underlying the results available to ensure full reproducibility?

Yes

Are the conclusions drawn adequately supported by the results?

Partly

Are sufficient details of methods and analysis provided to allow replication by others?

Yes

Reviewer Expertise:

Interdisciplinary approaches to the hegemonic agri-food system versus agroecological alternatives; Transdisciplinary methods for Participatory Action Research with practitioners on such issues.

I confirm that I have read this submission and believe that I have an appropriate level of expertise to confirm that it is of an acceptable scientific standard, however I have significant reservations, as outlined above.

# Response

**Published:** 2023-03-20

**Authors:** Ileana Iocola

**Affiliations:** Council for Agricultural Research and Economics, Research Centre for Agriculture and Environment (CREA-AA), Italy

We thank the referee for the careful review which helped improve the manuscript. Please find below a detailed point-by-point response to all comments.

**Q1) This paper has tensions among different emphases, both analytically and normatively. The Abstract speaks about agroecology for ‘more sustainable agriculture and food systems’, vaguely defined. More specifically, it mentions ‘a predominant trajectory of agroecology research prone to the transformation of the agri-food system’, at least as an aim. Elsewhere the paper distinguishes between transformational versus merely incremental changes, corresponding to the upper versus lower levels of Gliessman’s schema, respectively. That distinction potentially corresponds to interdisciplinary versus transdisciplinary research, the latter having greater involvement of non-production actors across the supply chain, especially value-chain actors. Although plausible, such correspondences ideally should be more sharply analysed in practice, beyond simply projects’ aims**.

 R1: we agree with the reviewer that agroecology for “more sustainable agriculture and food systems” is vaguely defined. In the new version of the abstract we replaced “ *Agroecology is increasingly seen as an important contribution for the development of more sustainable agriculture and food systems*” with “ *Redesigning the agri-food system through agroecology represents a common ambition among practitioners, activists and scholars* *to achieve environmental and socio-economic sustainability”. * However, in our article we did not mean to indicate any equivalence between transformational vs. merely incremental changes with inter- vs. trans-disciplinary research. In our work we affirm that since agroecology is characterized by a transformative approach, we can argue that agroecology research would be fully implemented from the level 3 of the Gliessman’s schema upwards. Consequently, given that agroecology requires transdisciplinary attitudes, we expect to find the application of this approach in all projects classified from the level 3 upwards. But this does not mean that a transdisciplinary approach cannot be implemented in participatory projects that aim to increase the sustainability of an agricultural system by reinforcing merely its efficiency. According to our opinion, transdisciplinary is “mandatory” from the level 3 upwards; for the other levels, despite not mandatory, its implementation is advisable.

**Q2) Those differences were investigated through a literature review of research projects and a questionnaire-survey of some. As an important finding, ‘Surveys reported a limited participation of value chain actors, while researchers, farmers, and their associations were the most involved’. This practical reality apparently contradicts many projects’ aims corresponding with upper levels of Gliessman’s schema, which emphasises short food-supply chains of a solidarity economy. The latter terms are absent from the analysis of projects, for unclear reasons: because they are absent from all the projects? It would be helpful to know what extent or type of downstream actors were involved, on the one hand, and what limited their involvement, on the other hand**.

 R2: Although not included in the keywords, solidarity economy was taken into account in the analysis of the projects. In fact “circular and solidarity economy” is one of the AE elements included in the ACT tool (see Table 2). Some projects addressed issues related to community values through different activities (i.e, development of a trademark for products from local breeds/varieties, policy recommendations for local and regional value chains, information/dissemination activities locally organised to encourage territorial and seasonal consumption); similarly, Community Supported Agriculture (CSAs) groups are sometimes partners of the same projects (i.e., Dynaversity). However, in many cases, a relative less attention to the development of local economies and value chain actors is granted by research projects. Indeed different projects, in addition to level 3, addressed other issues related to level 4 supporting topics related to co-creation and sharing of knowledge (i.e., farmers’ groups to share experiences, co-development of assessment tools, support for farmer-education networks and hubs, etc.). The suggestion to deeply know to what extent downstream actors are involved in the projects, and which type thereof, is very relevant. This information is often not easily obtainable for all projects in online documents and published papers. Our work helped to highlight this gap which necessarily has to be deepened with other more specific studies.

**Q3) In surveying EU research projects, the paper classifies them along the above lines by assigning percentages. EU projects have significant variations but rate more favourably (for upper levels) than international ones. As an important finding, the paper describes difficulties or limitations in implementing transformative aims, even through transdisciplinary research (Table 4). For example, sometimes ‘the mere involvement of non-academic actors is reduced to a consulting or informative process rather than a deep integration of knowledge because co-innovation and co-knowledge processes are not carried in all projects with LLs’ (Living Labs). Hence the statistical analysis can indicate only intentions, not necessarily practices. The paper could usefully identify projects which substantively fulfilled the more ambitious aims (or did not) and analyse how/why.**

 R3: All the identified research projects fulfilled their specific research objectives, but often, more ambitious general aims refer to a more in-depth change that occurs in the systems within a broader time frame. Table 4 is only related to the survey for researchers involved in agroecology and not to the projects. However, the implementation of transformative aims and transdisciplinary approaches are also cited as challenges by research project coordinators (Table 3). As correctly reported by the reviewer, our work has also highlighted that research projects that aim to redesign farm and agri-food systems are sometimes carrying out by implementing a non-genuine transdisciplinary approach, as they tend to consult local actors throughout the phases of the research, especially at transnational level. However, through the survey, we were able to intercept not only the intentions, but also how the research project coordinators perceive the contribution of their projects to agroecology issues, and some elements of practice such as how the scientific activities are performed in the projects, how the projects support co-learning and co-creation, difficulties and limitations encountered in the implementation of the agroecological research actions. We hadn’t the opportunity to deeply analyse the wide body of the results of the research projects (some of them still on-going) and their impacts. However, we are confident to have captured the main outcomes we were interested in. Our study has highlighted potential gaps and areas that need further analysis. As the reviewer suggests, it would be appropriate to deepen the study especially for those projects finished at least 3 years ago to investigate in detail the impacts on the real - (work) life of the non-academic beneficiaries. We have inserted a sentence that goes in this direction in the updated version of the manuscript:

*“In fact, according to the percentages of the ACT categories, in both surveys researchers implementing an approach of re-design of the farm/agroecosystem and the whole agri-food system represent the majority. However, it would be useful to extend the study to evaluate whether those research projects characterized by a trasformative approach were then actually able to have an impact and to make changes in the system beyond their temporal duration."*

**Q4)** **As another parameter, the paper does a social network analysis (SNA) of EU projects, identifying actors’ interconnections across projects and countries. The results indicate significant differences among projects. It remains unclear how these relate to the above distinction between incremental versus transformative aims, likewise between interdisciplinary versus transdisciplinary research. So the SNA lacks clear relevance to the overall argument. The paper could clarify whether this is a loose end warranting further analysis and perhaps more information**.

 R4: SNA is not related to the distinction between incremental versus transformative and it does not identify the interconnection of actors across the projects and countries. SNA aims to understand connections among European countries involved in agroecology research in order to identify the most powerful, influential countries and the poorly involved ones. In fact, the general aim of our work is to support, through research-based evidence, the proper design (including aims, methods, approaches to implement in the research and countries to involve) of the new research programmes related to agroecology within the Horizon framework to foster agroecology in Europe. A proper involvement of a variety of countries in those research programmes can contribute to align the different national strategies and to support research activities in agroecology within a common vision. In the updated version of our manuscript, we have changed the introduction (anticipating some concepts reported in the discussions of the work) and the objectives of our work to better highlight these aspects. Specifically, we added the following sentences removing them from the discussion:

 “ *Agroecology is also increasingly gaining attention in the context of funding research programmes as it is now explicitly mentioned in a clear and evident way within both the Horizon Europe framework (2021-2027) and the new partnership “Accelerating farming systems transition: agroecology living labs and research infrastructures”. Once more fully integrated in these research programmes, funding needs to be properly designed to ensure it promotes the transformative paradigm of agroecology across Europe. With the aim to align the different countries under this common transformative vision, participation and access to this funding should be guaranteed and promoted by the research programmes to all countries especially those less involved in agroecology or having different agricultural goals or interests. Previous studies based on network theory (Enger and Gulbrandsen, 2020; Protogerou et al., 2013) reported that few countries access to the projects and related resources within European Framework programmes. An investigation of the degree of involvement of the different countries in European research projects related to agroecology as well as their networking and relations can help to highlight useful insights for the funders’ perspective.*”. Moreover we have reformulated the aims (ii) of our work. Now the objectives (reformulated also considering the comments of the second reviewer) are the following: *“With the main objective to support the design of funding research programmes within the Horizon Europe framework and the new partnership on agroecology, this study mapped research projects related to agroecology funded by the European Union and/or co-funded by Member States to: i) characterize this research in terms of implementation of agroecology elements and evaluate if this current agroecology research really contributes to create transformative alternatives to the current agri-food regime; ii) understand connections among European countries participating to this research and identify the most powerful and influential countries investing in agroecology research and the less active ones, whose involvement should instead be promoted; and iii) provide recommendations for future research agendas within these funding research programmes to better strengthen agroecology and its transformative role in Europe.”*

**Q5) The Conclusion makes a cryptic comment, ‘the boundaries between the productivist and the transformative models are becoming blurred’. By contrast, the Introduction paragraph 2 implies that there is a clear boundary between different agendas appropriating agroecological techniques. So the ‘blurred boundaries’ remain unclear — in their own right, in relation to the empirical results and in relation to research agendas. If true, then it might complicate the paper’s key distinctions, as summarised in my first paragraph.**

 R5: We agree with the reviewer. This sentence is cryptic and its meaning can be confused. The boundaries between the conformative and the transformative models are becoming blurred not because it is not possible to distinguish them. In times of a crisis such as the one we are facing with the Ukraine conflict, the idea is gaining ground that agroecology cannot feed the world. "Green" concepts related to the dominant regime (eco-efficiency, sustainable intensification, genetically modified organisms for integrated pest management, etc.) rebecome en vogue. And in this time, these concepts can gain ground and justify their application also in agroecology thus blurring the boundaries. To be less cryptic, in the updated version of the manuscript we have replaced the sentence, “ *Nevertheless, today more than ever, given that the boundaries between the productivist and the transformative models are becoming blurred, it is necessary to ensure that the transformative role of agroecology be made more decipherable and visible*” with “ *Nevertheless, in times of a crisis such as the one we are facing with the Ukraine conflict, “green” concepts related to the dominant regime can rebecome en vogue, and justify their application also in agroecology. Today more than ever, it is therefore necessary to ensure that the transformative role of agroecology be made more decipherable and visible”*

**Q6) The Conclusion makes several recommendations for how structural changes in EU research funding could better facilitate research for a transformational agroecology. Yes, certainly ‘strengthening transdisciplinary research and increasing complexity’, especially in expecting greater involvement by value-chain actors. However, larger budgets and longer duration would impose greater administrative burdens in assembling consortia and managing projects, thus precluding or marginalising potential partnerships that may have important contributions. Indeed, when I briefly represented a pro-agroecology scientists’ consortium in EU research discussions, I had a mandate to advocate more projects with smaller budgets.**

 R6: The effectiveness of big and largely funded projects to provide quality outcomes for agroecology is widely questioned and the feedbacks obtained by the scientists and the coordinators we interviewed were consistent with this vision. The promotion of small projects is also considered a way to more effectively and closely involve a wider audience of actors not necessarily or poorly familiar with the administrative procedures requested by the research funding procedures, the administrative burdens in assembling large consortia and the difficulties of managing big projects. Also, in low sized projects, territorial and conceptual boundaries can be more sharply definable, enhancing the peer participation of different actors, increasing the co-creation potential and mitigating the risk of not-properly tailored research actions. However, we also recorded tensions in favour of complexity, interpreted as a way to avoid over-simplification of processes and shallow interactions that the straightforward and linear approaches might imply. In our opinion, these two lines of thinking are not divergent and should be simultaneously considered. We highlighted the outcomes obtained in this regard in our recommendations to advice the research programme planners to consider the issue of project size not only in relation to the budget available, but also considering the project topic(s), the scale of intervention and the expected impact. In the revised version, we expanded the recommendation #10 to be more explicit and possibly exhaustive; now the text is the following:

 “ *Frame research programmes in a way that does not allow small projects whose results might be too simplified, as well as very large one that cannot be efficiently managed. Envision projects dimension also considering the topic(s), the scale of intervention and the expected impact*."

**Q7) Some recommendations here seem like an administrative fix for more fundamental limitations that need different solutions in transforming research cultures and institutions. In particular, value chains potentially shape production choices, methods and outlets -- but may seem less ‘scientific’ issues for justifying research. This relates to the above lacuna as regards the limited value-chain actors so far involved, even in projects espousing transdisciplinary research. The paper could better reflect on those limitations and means to overcome them for transformative agendas.**

 R7: We thank the reviewer for this very useful suggestion. We have added some sentences to the manuscript to better express these concepts. Specifically, in the Policy recommendations paragraph, after, “ *To address the social challenges posed by agroecology (especially those related to level four and five), research should involve a greater number of actors from the entire agri-food system, in particular those who are currently less represented such as upstream and downstream value chain actors”* *we have added* *the following sentences*: *"* *In order to achieve this goal, research should identify suitable avenues and competences to not only redesign production but also processing, retailing and consumption models based on agroecological principles within a food system approach. In particular, as value chains potentially shape production choices, methods and outlets, agroecology research should explore ways to better integrate downstream actors in reconfiguring value chains within alternative food systems, with recognizable products and processing practices, democratising the economic prospects of the agroecological transition".*

 Furthermore, in the conclusion, after “ *Mainly, our outcomes suggest fostering the transformative role of research in agroecology by considering the whole agri-food system together with its various actors*” we added “ *exploring new business and governance models that facilitate integration with downstream sectors and consumers*”
